# Reduced Efficacy of d-Amphetamine and 3,4-Methylenedioxymethamphetamine in Inducing Hyperactivity in Mice Lacking the Postsynaptic Scaffolding Protein SHANK1

**DOI:** 10.3389/fnmol.2018.00419

**Published:** 2018-11-16

**Authors:** A. Özge Sungur, Tobias M. Redecker, Elena Andres, Wiebke Dürichen, Rainer K. W. Schwarting, Adriana del Rey, Markus Wöhr

**Affiliations:** ^1^Behavioral Neuroscience, Experimental and Biological Psychology, Philipps University of Marburg, Marburg, Germany; ^2^Center for Mind, Brain and Behavior, Philipps University of Marburg, Marburg, Germany; ^3^Research Group Immunophysiology, Division of Neurophysiology, Institute of Physiology and Pathophysiology, Philipps University of Marburg, Marburg, Germany

**Keywords:** ecstasy, MDMA, dopamine, noradrenaline, norepinephrine, serotonin

## Abstract

Genetic defects in the three SH3 and multiple ankyrin repeat domains (SHANK) genes (*SHANK1, SHANK2*, and *SHANK3*) are associated with multiple major neuropsychiatric disorders, including autism spectrum disorder (ASD), schizophrenia (SCZ), and bipolar disorder (BPD). Psychostimulant-induced hyperactivity is a commonly applied paradigm to assess behavioral phenotypes related to BPD and considered to be the gold standard for modeling mania-like elevated drive in mouse models. Therefore, the goal of our present study was to test whether *Shank1* plays a role in the behavioral effects of psychostimulants and whether this is associated with genotype-dependent neurochemical alterations. To this aim, male and female null mutant *Shank1^-/-^* mice were treated with d-amphetamine (AMPH; 2.5 mg/kg) and 3,4-methylenedioxymethamphetamine (MDMA, commonly known as ecstasy; 20 mg/kg), and psychostimulant-induced hyperactivity was compared to heterozygous *Shank1^+/-^* and wildtype *Shank1^+/+^* littermate controls. Results show that *Shank1^-/-^* mice display reduced psychostimulant-induced hyperactivity, although psychostimulants robustly stimulated locomotor activity in littermate controls. *Shank1* deletion effects emerged throughout development, were particularly prominent in adulthood, and seen in response to both psychostimulants, i.e., AMPH and MDMA. Specifically, while AMPH-induced hyperactivity was reduced but still detectable in *Shank1^-/-^* mice, MDMA-induced hyperactivity was robustly blocked and completely absent in *Shank1^-/-^* mice. Reduced efficacy of psychostimulants to stimulate hyperactivity in *Shank1^-/-^* mice might be associated with alterations in the neurochemical architecture in prefrontal cortex, nucleus accumbens, and hypothalamus. Our observation that psychostimulant-induced hyperactivity is reduced rather than enhanced in *Shank1^-/-^* mice clearly speaks against a behavioral phenotype with relevance to BPD. Lack of BPD-like phenotype is consistent with currently available human data linking mutations in *SHANK2* and *SHANK3* but not *SHANK1* to BPD.

## Introduction

Genetic defects in the three SH3 and multiple ankyrin repeat domains (SHANK) genes (*SHANK1, SHANK2*, and *SHANK3*) are associated with multiple major neuropsychiatric disorders, including autism spectrum disorder (ASD), schizophrenia (SCZ), and bipolar disorder (BPD; Guilmatre et al., 2014; Bourgeron, 2015; de la Torre-Ubieta et al., 2016). Early genetic studies provided compelling evidence implicating *SHANK* mutations in the whole spectrum of ASD (Durand et al., 2007; Moessner et al., 2007; Gauthier et al., 2009; Berkel et al., 2010; Pinto et al., 2010; Sato et al., 2012). However, there is increasing evidence suggesting that *SHANK* mutations play a prominent role in other neuropsychiatric disorders as well. For instance, Fromer et al. (2014) reported a *de novo SHANK1* frameshift mutation in a SCZ patient, and Lennertz et al. (2012) found that a *SHANK1* promotor variant is associated with reduced auditory working memory capacity in SCZ patients. Mutations in *SHANK2* and *SHANK3* have likewise been described in individuals with SCZ (Gauthier et al., 2010; Peykov et al., 2015; Homann et al., 2016). Moreover, a duplication in *SHANK2* has been reported for a BPD patient (Noor et al., 2014) and BPD has been diagnosed also in individuals with the Phelan-McDermid 22q13 deletion syndrome lacking *SHANK3* (Sovner et al., 1996; Willemsen et al., 2011; Denayer et al., 2012; Verhoeven et al., 2012, 2013; Vucurovic et al., 2012). It thus appears that *SHANK* mutations are shared across multiple neuropsychiatric disorders similar to other genetic risk factors, pointing to extensive pleiotropy and challenging the biological validity of existing diagnostic approaches (Zhu et al., 2014; Geschwind and Flint, 2015; O’Donovan and Owen, 2016). However, the neurobiological mechanisms underlying the pleiotropic effects of *SHANK* mutations are not well understood.

The three *SHANK* genes code for several mRNA splice variants and generate multiple protein isoforms (Ting et al., 2012; Sala et al., 2015). Members of the SHANK protein family have five conserved protein domains through which they assemble into large molecular platforms in the postsynaptic density (PSD) at excitatory glutamatergic synapses. More than 30 synaptic proteins have been reported to form interactions with SHANK protein family members. As master scaffolding proteins, SHANKs anchor glutamate receptors and link them to the actin cytoskeleton and postsynaptic signaling pathways. They are thus strongly involved in several synaptic functions, including spine morphogenesis, synapse formation, glutamate receptor trafficking, and activity-dependent neuronal signaling.

Several *Shank* mouse models were generated during the last decade (Jiang and Ehlers, 2013; Yoo et al., 2013; Schmeisser and Verpelli, 2016). While *Shank2* and *Shank3* mutant mice have been extensively characterized (Peça et al., 2011; Wang et al., 2011; Schmeisser et al., 2012; Won et al., 2012), comparatively little is known about *Shank1* mutant mice (Sungur et al., 2018). Hung et al. (2008) generated *Shank1* mutant mice via deletion of exons 14 and 15 encoding almost the entire PDZ domain. This approach resulted in a complete knockout of all SHANK1 protein isoforms. Behavioral studies employing *Shank1^-/-^* mice almost exclusively focused on ASD-related behavioral phenotypes and cognitive deficits with relevance to intellectual disability (Silverman et al., 2010). Specifically, Hung et al. (2008) performed an extensive set of cognitive tasks and found that *Shank1^-/-^* mice display impaired contextual but intact cued fear memory, and enhanced acquisition but impaired retention of spatial memory, possibly resembling the aberrant cognitive phenotype present in some ASD cases. Extending these findings, we recently showed that *Shank1^-/-^* mice are severely impaired in novel object recognition memory and that this deficit is associated with increased expression of brain-derived neurotrophic factor BDNF in the hippocampus, possibly due to epigenetic modifications, as indicated by enrichment of histone H3 acetylation at the *Bdnf* promoter1 in *Shank1^-/-^* mice (Sungur et al., 2017). Consistent with ASD-related behavioral phenotypes, we further showed that *Shank1^-/-^* mice show prominent vocal and olfactory communication deficits (Wöhr et al., 2011; Wöhr, 2014; Sungur et al., 2016), together with a moderate increase in self-grooming behavior (Sungur et al., 2014) and mild alterations in social behavior (Silverman et al., 2011; Sungur et al., 2017).

However, because mutations in *SHANK* genes are associated with multiple major neuropsychiatric disorders, including SCZ and BPD besides ASD, we reasoned that a more comprehensive characterization of *Shank1^-/-^* mice might provide novel insights into the complex role *SHANK1* appears to play. A commonly applied paradigm to assess behavioral phenotypes related to SCZ and BPD in mouse models is psychostimulant-induced hyperactivity (Kato et al., 2007; Young et al., 2011). Psychostimulants, such as d-amphetamine (AMPH), can provoke mania-like symptoms in healthy subjects and exacerbate symptoms or induce a manic episode in BPD patients (Meyendorff et al., 1985; Peet and Peters, 1995; Hasler et al., 2006). AMPH-induced hyperactivity is thus considered to be the gold standard for modeling mania-like elevated drive in rodents (Berggren et al., 1978; Gould et al., 2001; Pereira et al., 2014). While AMPH affects primarily the catecholaminergic system and results in increased extracellular dopamine (DA) and noradrenaline (NA) concentrations through direct interaction with DA and NA transporters, other psychostimulants, such as 3,4-methylenedioxymethamphetamine (MDMA; commonly known as ecstasy), differ in their mode of action by having particularly strong effects on serotonin (5-HT) in addition (Sulzer et al., 2005; Hutson et al., 2014). Importantly, *Shank3* overexpression was linked to increased locomotor activity in mice and Han et al. (2013) found that an acute injection of AMPH aggravated the hyperactivity of *Shank3* overexpressing mice to a greater extent than in controls. Moreover, Pappas et al. (2017) recently generated *Shank2* mutant mice lacking exon 24 (Δ24) and found that *Shank2^Δ24-/-^* mice display elevated levels of locomotor activity, which could be normalized by lithium and valproate treatment, but were further enhanced by AMPH administration. The augmentation with AMPH was more prominent in *Shank2^Δ24-/-^* mice than in wildtype *Shank2^Δ24+/+^* controls (Pappas et al., 2017).

Here, we therefore asked whether *Shank1* plays a role in the behavioral effects of psychostimulants and whether this is associated with genotype-dependent neurochemical alterations. Firstly, at the behavioral level, we assessed psychostimulant-induced hyperactivity in *Shank1^-/-^* null mutant mice in comparison to heterozygous *Shank1^+/-^* and wildtype *Shank1^+/+^* littermate controls across development. To this aim, juvenile and adult subject mice were treated with the psychostimulant AMPH primarily targeting DA and NA transporters, and their locomotor activity was assessed in an open field. To assess specificity and to evaluate generalizability, subject mice were further treated with the psychostimulant MDMA, which has particularly strong effects on the 5-HT transporter. Secondly, at the neurobiological level, we analyzed DA, NA, and 5-HT neurotransmitter concentrations together with their precursors and metabolites in relevant brain structures, namely prefrontal cortex, nucleus accumbens, and hypothalamus.

## Materials and Methods

### Animals and Housing

Juvenile and adult *Shank1^-/-^* null mutant mice with a targeted replacement of exons 14 and 15 encoding almost the entire PDZ domain were compared to *Shank1^+/-^* heterozygous and *Shank1^+/+^* wildtype littermate control mice. Mice were obtained from mutant lines originally generated by Hung et al. (2008) on two independent background strains: C57BL/6J and 129SvJae. As high mortality rates were obtained in the C57BL/6J background strain and very low locomotion in the 129SvJae background strain (Hung et al., 2008; Silverman et al., 2011), the two lines were crossed for at least three generations to produce a mixed C57BL/6J/129SvJae background for the *Shank1* mutation. This mixed background was maintained since then and used in the present study, consistent with other studies focusing on this *Shank1* mutant (Hung et al., 2008; Wöhr et al., 2011; Silverman et al., 2011; Sungur et al., 2014, 2017, 2018; Wöhr, 2014). Using a heterozygous breeding protocol, *Shank1^+/-^* males and females were bred in a conventional vivarium at the Biomedical Research Center of the Philipps-University of Marburg, Germany. Approximately 2 weeks after pairing for breeding, females were individually housed and inspected daily for pregnancy and delivery. The day of birth was considered as postnatal day (PND) 0. After weaning on PND21, mice were socially housed in groups of 2–6 with same-sex partners in polycarbonate Makrolon type III IVC cages (L × W × H: 420 × 265 × 180 mm, 825 cm^2^; Ehret, Emmendingen, Germany). Bedding and a wooden board were provided in each cage. Standard rodent chow and water were available ad libitum. The colony room was maintained on a 12:12 light/dark cycle (lights on: 06:00h) at approximately 22°C and 40–50% humidity. Pups were identified by paw tattoo, using non-toxic animal tattoo ink (Ketchum permanent Tattoo Inks green paste, Ketchum Manufacturing Inc., Brockville, ON, Canada). The ink was inserted subcutaneously through a 30 gauge hypodermic needle tip into the center of the paw. For genotyping, mouse tail snips were collected by dissecting ∼0.3 cm of tail between PND3-12. Genotyping was performed as described previously (Sungur et al., 2014). All procedures were approved by the ethical committee of the Regierungspräsidium Gießen, Germany [file reference: V54–19c 20 15 (1) MR20/35 Nr. 20/2010 and V54–19c 20 15 h 01 MR20/35 Nr. G 30/2018].

### Experimental Design

For testing whether *Shank1* plays a role in the behavioral effects of psychostimulants, male and female null mutant *Shank1^-/-^* mice were treated with AMPH (2.5 mg/kg) and MDMA (20 mg/kg), and psychostimulant-induced hyperactivity was compared to heterozygous *Shank1^+/-^* and wildtype *Shank1^+/+^* littermate controls. AMPH-induced hyperactivity was assessed in juvenile (*Shank1^+/+^*: *N* = 15; *Shank1^+/-^*: *N* = 20; *Shank1^-/-^*: *N* = 16) and adult (*Shank1^+/+^*: *N* = 24; *Shank1^+/-^*: *N* = 28; *Shank1^-/-^*: *N* = 28) mice. MDMA-induced hyperactivity was determined in adulthood only (*Shank1^+/+^*: *N* = 14; *Shank1^+/-^*: *N* = 19; *Shank1^-/-^*: *N* = 14). Drug doses are expressed as salt and were determined based on previous studies reporting psychostimulant-induced hyperactivity (Risbrough et al., 2006; Young et al., 2010).

### Drug Treatment

d-amphetamine sulfate (AMPH; Sigma–Aldrich, MO, United States) and 3,4-Methylendioxy-*N*-methylamphetamin (MDMA; Lipomed, Switzerland) were dissolved in 0.9% saline and administered intraperitoneally (i.p.) at volume of 1 ml/kg. As vehicle control, 0.9% saline was used. Injections were performed immediately prior to open field exposure for locomotor activity assessment.

### Open Field

Psychostimulant-induced hyperactivity was measured in an open field (50 cm × 50 cm × 35 cm; TSE Systems, Bad Homburg, Germany) under dim red light, according to a previously established protocol (Natusch and Schwarting, 2010). Individual mice were placed randomly in one of the corners of the open field and were allowed to explore the apparatus for 45 min on three consecutive days. On the first day (baseline), mice were habituated to the open field. On the second day (saline), mice were administered saline. On the third day (treatment), mice were administered AMPH or MDMA. Distance traveled, rearing behavior, and the time spent in the center (30 cm × 30 cm) were automatically collected using the TSE VideoMot2 analyzer software (TSE Systems, Bad Homburg, Germany). Circling behavior, i.e., turning in a circular motion, was quantified by a trained observer.

### Tissue Preparation

Drug-naïve juvenile mice of both sexes were individually caged and sacrificed by cervical dislocation. Immediately after the brain was removed, prefrontal cortex, nucleus accumbens, and hypothalamus were dissected using a special brain matrix, designed by Dr. J. Wildmann (Institute for Physiology and Pathophysiology, Philipps-University, Marburg). The different regions were divided into right and left parts, quickly frozen on dry ice, and stored at -80°C until used for neurotransmitter determination.

### Neurotransmitter Determination

The concentration of catecholaminergic and indolaminergic neurotransmitters, metabolites, and precursors were measured using high performance liquid chromatography (HPLC), as previously described (Roggero et al., 2016). Briefly, frozen brain samples were homogenized in 0.4 M HClO_4_ by sonication and 10 μl of the supernatant was injected for the analysis. The HPLC system used consists of a reversed-phase C-18 chromatography column (Supelco, Sigma–Aldrich, St. Louis, MO, United States) as stationary phase and an aqueous eluent containing 10% acetonitrile as mobile phase. Besides the neurotransmitters DA, NA, and 5-HT, the precursors tyrosine (Tyr) and tryptophan (Trp) as well as the metabolites dehydroxyphenylacetic acid (DOPAC), 4-hydroxy-3-methoxy-phenylglycol (MHPG), and 5-hydroxy-indol-acetic acid (5-HIAA) were quantified in each sample. Quantification was done by peak height evaluation or by area integration using Chromeleon software version 6.08 from Dionex (Sunnyvale, CA, United States).

### Statistical Analysis

Distance traveled in the open field was analyzed using analyses of variance (ANOVAs) for repeated measurements with genotype and sex as between-subject factors and test day or test minute as within-subject factor. Comparison between test days for individual genotypes was performed by calculating paired samples *t*-tests. Neurotransmitter concentrations were analyzed using one-way ANOVAs with genotype as between-subject factor. For each neurotransmitter, values that were smaller than the lower quartile minus three times the interquartile range or larger than the upper quartile plus three times the interquartile range were considered as outliers and excluded from analysis. When appropriate, ANOVAs were followed by LSD *post hoc* analysis for comparing genotypes. A *p*-value of < 0.050 was considered statistically significant.

## Results

### AMPH-Induced Hyperactivity

**FIGURE 1:**
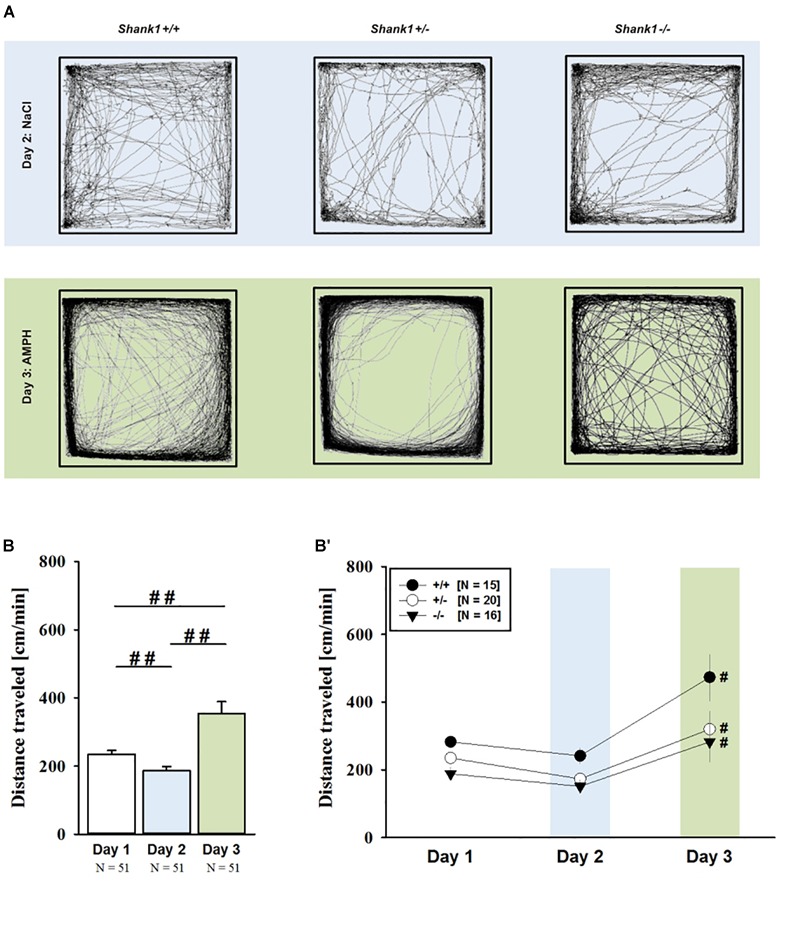
AMPH-induced hyperactivity in juvenile *Shank1* mice. **(A)** Exemplary figure depicting the locomotor activity pattern of individual juvenile *Shank1^+/+^, Shank1^+/-^*, and *Shank1^-/-^* mice injected with saline (NaCl) on day 2 (upper panel) and with AMPH on day 3 (lower panel); measured over 45 min. **(B)** Bar graph depicting the distance traveled by all genotypes during baseline testing (white bar), following saline administration (blue bar), and after AMPH treatment (green bar). **(B′)** Line graph depicting the distance traveled by *Shank1^+/+^* (black circle), *Shank1^+/-^* (white circle), and *Shank1^-/-^* (black triangle) over the three consecutive test days. Data are presented as means + SEM or means ± SEM. ^##^*p* < 0.001 **(B)**, ^#^*p* < 0.05 vs. day 2 **(B′)**.

**FIGURE 2:**
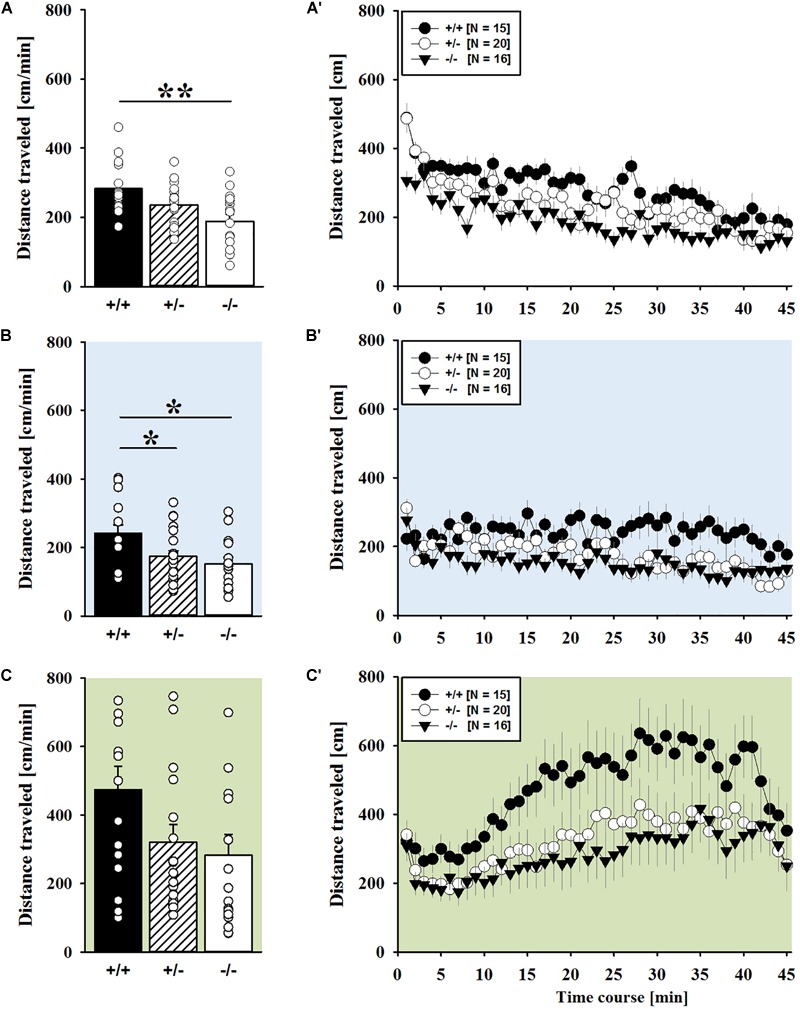
AMPH-induced hyperactivity in juvenile *Shank1* mice. **(A–C)** Bar graphs and **(A′–C′)** line graphs depicting the distance traveled by *Shank1^+/+^* (black bar/black circle), *Shank1^+/-^* (striped bar/white circle), and *Shank1^-/-^* (white bar/black triangle). Locomotor activity was compared during baseline testing **(A,A′)**, following saline administration **(B,B′)**, and after AMPH treatment **(C,C′)**. Data are presented as means + SEM or means ± SEM. ^∗^*p* < 0.05, ^∗∗^*p* < 0.001.

AMPH administration induced hyperactivity in juvenile mice (*F*_2,90_ = 20.840, *p* < 0.001), as evidenced by representative locomotor activity patterns in response to AMPH on test day 3 in comparison to saline on day 2 (Figure 1A). Importantly, there was also an overall effect of genotype (*F*_2,45_ = 4.766, *p* = 0.013; day × genotype: *F*_4,90_ = 0.775, *p* = 0.545), but no general sex effect and no sex interaction with AMPH treatment (*F*_1,45_ = 0.139, *p* = 0.711; sex × genotype: *F*_2,45_ = 0.032, *p* = 0.968; day × sex: *F*_2,90_ = 0.198, *p* = 0.821; day × sex × genotype: *F*_4,90_ = 0.428, *p* = 0.788). A comparison between test days revealed that the distanced traveled following AMPH treatment was clearly higher than the day before in response to saline (*p* < 0.001; Figure 1B). This difference held true for individual genotypes (*Shank1^+/+^*: *t*_14_ = 3.787, *p* = 0.001, one-tailed; *Shank1^+/-^*: *t*_19_ = 2.953, *p* = 0.008; *Shank1^-/-^*: *t*_15_ = 2.495, *p* = 0.025; Figure 1B′). When comparing locomotor activity between individual genotypes separately for each test day, evidence for genotype-dependent differences in the distance traveled was obtained under baseline conditions (*F*_2,48_ = 6.619, *p* = 0.003; Figure 2A) and in response to saline (*F*_2,48_ = 5.076, *p* = 0.010; Figure 2B). Specifically, on day 1, *Shank1^+/+^* mice displayed slightly higher levels of locomotor activity than *Shank1^-/-^* mice (*p* = 0.001) but did not differ significantly from *Shank1^+/-^* mice (*p* = 0.059). Similarly, on day 2, *Shank1^+/+^* mice engaged more in locomotor activity than *Shank1^-/-^* (*p* = 0.004) and *Shank1^+/-^* mice (*p* = 0.019). Locomotor activity displayed by *Shank1^+/-^* and *Shank1^-/-^* mice did not differ from each other under baseline conditions (*p* = 0.059) and in response to saline (*p* = 0.428). Although there was only a trend for a genotype effect on AMPH-induced hyperactivity on the third test day and the overall level of locomotor activity was not significantly affected by *Shank1* deletion (*F*_2,48_ = 2.602, *p* = 0.085), the temporal response pattern differed between genotypes (genotype: *F*_2,45_ = 2.186, *p* = 0.124; time × genotype: *F*_88,1980_ = 1.278, *p* = 0.045; Figure 2C). After about 10 min with similar locomotor activity levels in all genotypes, *Shank1^+/+^* mice displayed a strong increase in locomotor activity and maintained high activity levels for about 30 min, whereas the AMPH-induced increase was less prominent in *Shank1^+/-^* and *Shank1^-/-^* mice (Figures 2A′–C′).

Of note, the time spent in the center of the open field was also affected and changed across test days (*F*_2,90_ = 33.804, *p* < 0.001). There were overall effects of genotype (*F*_2,45_ = 9.573, *p* < 0.001; day × genotype: *F*_4,90_ = 2.774, *p* = 0.032) and sex (*F*_1,45_ = 7.393, *p* = 0.009; sex × genotype: *F*_2,45_ = 0.091, *p* = 0.914), but no sex interaction with AMPH treatment (day × sex: *F*_2,90_ = 0.352, *p* = 0.704; day × sex × genotype: *F*_4,90_ = 0.188, *p* = 0.944). When comparing the time spent in the center between individual genotypes separately for each test day, prominent genotype differences were detected across all three test days. Specifically, under baseline conditions, genotypes differed from each other (*F*_2,48_ = 9.360, *p* < 0.001; Supplementary Figure 1A) and *Shank1^+/+^* mice spent more time in the center than *Shank1^+/-^* (*p* = 0.018) and *Shank1^-/-^* mice (*p* < 0.001), with the latter also differing from each other (*p* = 0.037). Similarly, center time differed between genotypes in response to saline on day 2 (*F*_2,48_ = 4.388, *p* = 0.018; Supplementary Figure 1B), with *Shank1^+/+^* mice again spending more time in the center than *Shank1^+/-^* (*p* = 0.015) and *Shank1^-/-^* mice (*p* = 0.010), but the latter not differing from each other (*p* = 0.774). A similar pattern was obtained also in response to AMPH on day 3 (*F*_2,48_ = 5.367, *p* = 0.008; Supplementary Figure 1C), with *Shank1^+/+^* mice spending more time in the center than *Shank1^+/-^* (*p* = 0.011) and *Shank1^-/-^* mice (*p* = 0.004), but the latter not differing from each other (*p* = 0.546).

**FIGURE 3:**
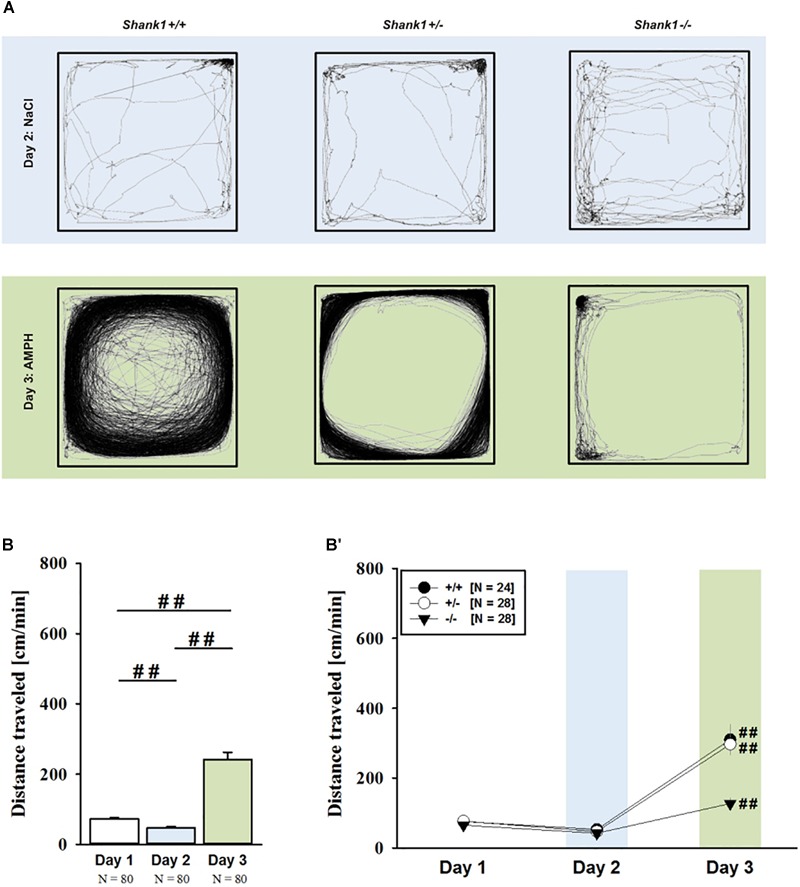
**(A)** AMPH-induced hyperactivity in adult *Shank1* mice. Exemplary figure depicting the locomotor activity pattern of individual adult *Shank1^+/+^, Shank1^+/-^*, and *Shank1^-/-^* mice injected with saline (NaCl) on day 2 (upper panel) and with AMPH on day 3 (lower panel); measured over 45 min. **(B)** Bar graph depicting the distance traveled by all genotypes during baseline testing (white bar), following saline administration (blue bar), and after AMPH treatment (green bar). **(B′)** Line graph depicting the distance traveled by *Shank1^+/+^* (black circle), *Shank1^+/-^* (white circle), and *Shank1^-/-^* (black triangle) over the three consecutive test days. Data are presented as means + SEM or means ± SEM. ^##^*p* < 0.001 **(B)**, ^##^*p* < 0.001 vs. day 2 **(B′)**.

**FIGURE 4:**
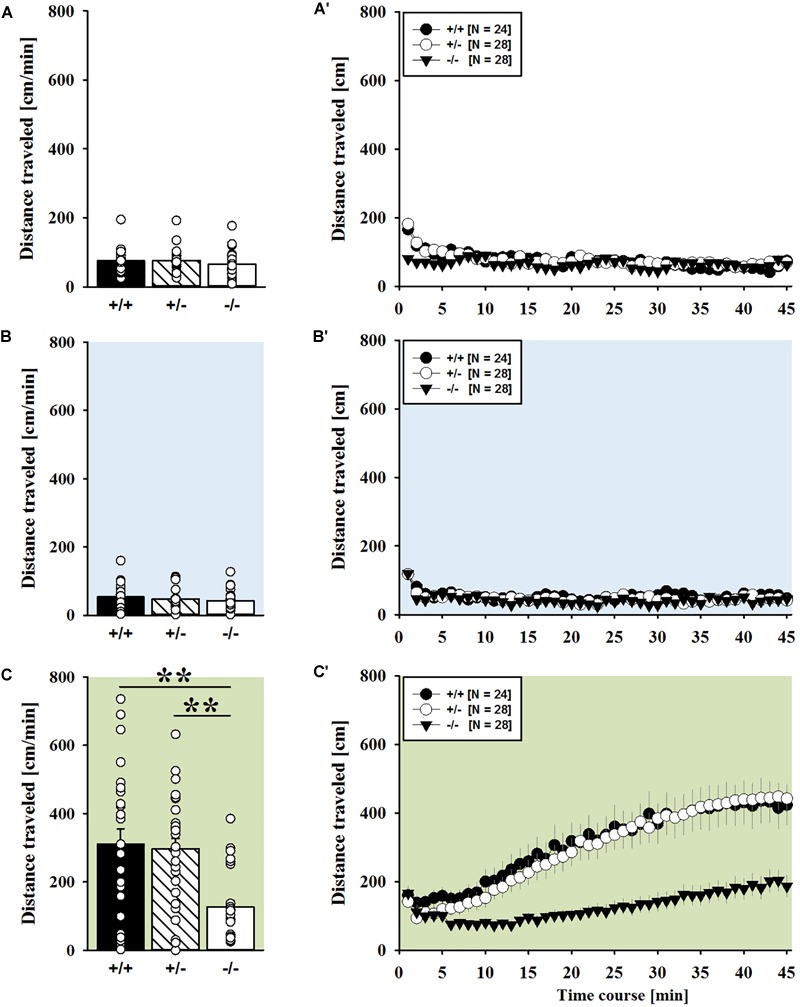
AMPH-induced hyperactivity in adult *Shank1* mice. **(A–C)** Bar graphs and **(A′–C′)** line graphs depicting the distance traveled by *Shank1^+/+^* (black bar/black circle), *Shank1^+/-^* (striped bar/white circle), and *Shank1^-/-^* (white bar/black triangle). Locomotor activity was compared during baseline testing **(A,A′)**, following saline administration **(B,B′)**, and after AMPH treatment **(C,C′)**. Data are presented as means + SEM or means ± SEM. ^∗∗^*p* < 0.001.

Consistent with the data obtained in juveniles, AMPH administration induced hyperactivity in adult mice (*F*_2,148_ = 107.888, *p* < 0.001), and this is similarly evidenced by representative locomotor activity patterns on test day 3 in comparison to saline on day 2 (Figure 3A). Again, there was a general genotype effect (*F*_2,74_ = 10.233, *p* < 0.001), while sex had no prominent impact and did not modulate AMPH effects in a genotype-dependent manner (*F*_1,74_ = 3.358, *p* = 0.064; sex × genotype: *F*_2,74_ = 0.349, *p* = 0.707; day × sex: *F*_2,148_ = 3.324, *p* = 0.039; day × sex × genotype: *F*_4,148_ = 0.957, *p* = 0.433). This was further supported by a comparison between test days that revealed clearly higher levels of locomotor activity following AMPH treatment than the day before in response to saline (*p* < 0.001; Figure 3B) and this difference held true for individual genotypes (*Shank1^+/+^*: *t*_23_ = 5.800, *p* < 0.001, one-tailed; *Shank1^+/-^*: *t*_27_ = 8.478, *p* < 0.001; *Shank1^-/-^*: *t*_27_ = 5.123, *p* < 0.001; Figure 3B′). Despite this general effect, however, AMPH responsivity in adult mice was strongly affected by *Shank1* deletion (day × genotype: *F*_4,148_ = 10.030, *p* < 0.001). While locomotor activity did not differ between genotypes under baseline conditions (*F*_2,77_ = 0.980, *p* = 0.380; Figure 4A) and no differences were evident in response to saline (*F*_2,77_ = 0.801, *p* = 0.453; Figure 4B), prominent genotype differences were evident following AMPH treatment. In contrast to juvenile mice, AMPH-induced hyperactivity was clearly dependent on genotype in adult mice (*F*_2,77_ = 10.449, *p* < 0.001). Specifically, in comparison to *Shank1^-/-^* mice, there was increased hyperactivity in *Shank1^+/+^* (*p* < 0.001) and *Shank1^+/-^* mice (*p* < 0.001) following AMPH treatment, whereas AMPH-induced hyperactivity did not differ between *Shank1^+/+^* and *Shank1^+/-^* mice (*p* = 0.768). This prominent genotype effect was also reflected in the temporal response pattern (genotype: *F*_2,77_ = 10.449, *p* < 0.001; time × genotype: *F*_88,3388_ = 6.004, *p* < 0.001; Figure 4C). After about 10 min with similar locomotor activity levels in all genotypes, *Shank1^+/+^* and *Shank1^+/-^* mice displayed a strong increase in locomotor activity and maintained high activity levels until the end of testing, whereas the AMPH-induced increase was very mild in *Shank1^-/-^* mice (Figures 4A′–C′).

Because *Shank1* deletion had prominent effects on the induction of locomotor activity following AMPH treatment in *Shank1^-/-^* mice but not *Shank1^+/-^* mice, we also tested whether rearing behavior was affected by genotype in a similar manner in adult mice. AMPH administration induced rearing behavior (*F*_2,148_ = 3.885, *p* = 0.023) and, alike locomotor activity, there was a general genotype effect on rearing behavior (*F*_2,74_ = 7.794, *p* < 0.001). Sex had no prominent impact and did not modulate AMPH effects (*F*_1,74_ = 0.179, *p* = 0.674; sex × genotype: *F*_2,74_ = 0.578, *p* = 0.536; day × sex: *F*_4,148_ = 0.544, *p* = 0.582; day × sex × genotype: *F*_4,148_ = 1.815, *p* = 0.129). Importantly, however, AMPH responsivity was again strongly affected by *Shank1* deletion (day × genotype: *F*_4,148_ = 2.926, *p* = 0.023). While rearing behavior did not differ between genotypes under baseline conditions (*F*_2,77_ = 1.163, *p* = 0.318; Supplementary Figure 2A) and no differences were evident in response to saline (*F*_2,77_ = 1.406, *p* = 0.251; Supplementary Figure 2B), prominent genotype differences were evident following AMPH treatment (*F*_2,77_ = 8.629, *p* < 0.001). Specifically, in comparison to *Shank1^+/+^* mice, there was reduced rearing behavior in *Shank1^+/-^* (*p* < 0.001) and *Shank1^-/-^* mice (*p* < 0.001) following AMPH treatment, whereas AMPH-induced rearing behavior did not differ between *Shank1^+/-^* and *Shank1^-/-^* mice (*p* = 0.788). This prominent genotype effect was also reflected in the temporal response pattern (genotype: *F*_2,77_ = 8.629, *p* < 0.001; time × genotype: *F*_88,3388_ = 2.040, *p* < 0.001; Supplementary Figure 2C). After about 10 min with similar levels of rearing behavior in all genotypes, only *Shank1^+/+^* mice displayed a strong increase in rearing behavior and maintained high rearing levels until the end of testing, whereas the AMPH-induced increase was very mild in *Shank1^+/-^* and *Shank1^-/-^* mice (Supplementary Figures 2A′–C′).

Of note, the time spent in the center of the open field was also affected and changed across test days (*F*_2,148_ = 30.879, *p* < 0.001), but no genotype-dependent differences were obtained (*F*_2,74_ = 0.998, *p* = 0.373; day × genotype: *F*_4,148_ = 1.579, *p* = 0.183), with sex having also no impact (*F*_1,74_ = 0.270, *p* = 0.605; sex × genotype: *F*_2,74_ = 0.138, *p* = 0.871; day × sex: *F*_2,148_ = 1.543, *p* = 0.217; day × sex × genotype: *F*_4,148_ = 0.660, *p* = 0.621). When comparing genotypes on individual test days, no differences were observed under baseline conditions (*F*_2,77_ = 0.573, *p* = 0.566; Supplementary Figure 3A) and in response to saline on day 2 (*F*_2,77_ = 2.590, *p* = 0.082; Supplementary Figure 3B), whereas genotypes differed from each other in response to AMPH on day 3 (*F*_2,77_ = 4.318, *p* = 0.017; Supplementary Figure 3C). Specifically, *Shank1^+/-^* mice spent less time in the center than *Shank1^+/+^* mice (*p* = 0.005) but not than *Shank1^-/-^* mice (*p* = 0.055), with the latter also not differing from each other (*p* = 0.324).

### MDMA-Induced Hyperactivity

**FIGURE 5:**
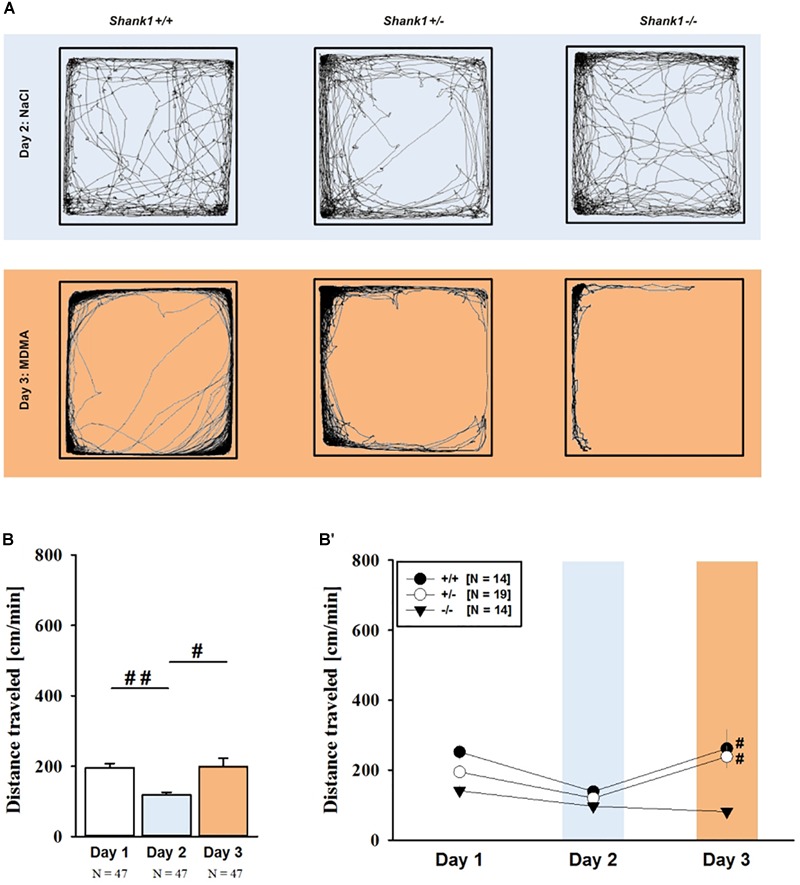
MDMA-induced hyperactivity in adult *Shank1* mice. **(A)** Exemplary figure depicting the locomotor activity pattern of individual adult *Shank1^+/+^, Shank1^+/-^*, and *Shank1^-/-^* mice injected with saline (NaCl) on day 2 (upper panel) and with MDMA on day 3 (lower panel); measured over 45 min. **(B)** Bar graph depicting the distance traveled by all genotypes during baseline testing (white bar), following saline administration (blue bar), and after MDMA treatment (orange bar). **(B′)** Line graph depicting the distance traveled by *Shank1^+/+^* (black circle), *Shank1^+/-^* (white circle), and *Shank1^-/-^* (black triangle) over the three consecutive test days. Data are presented as means + SEM or means ± SEM. ^#^*p* < 0.05, ^##^*p* < 0.001 **(B)**, ^#^*p* < 0.05 vs. day 2 **(B′)**.

**FIGURE 6:**
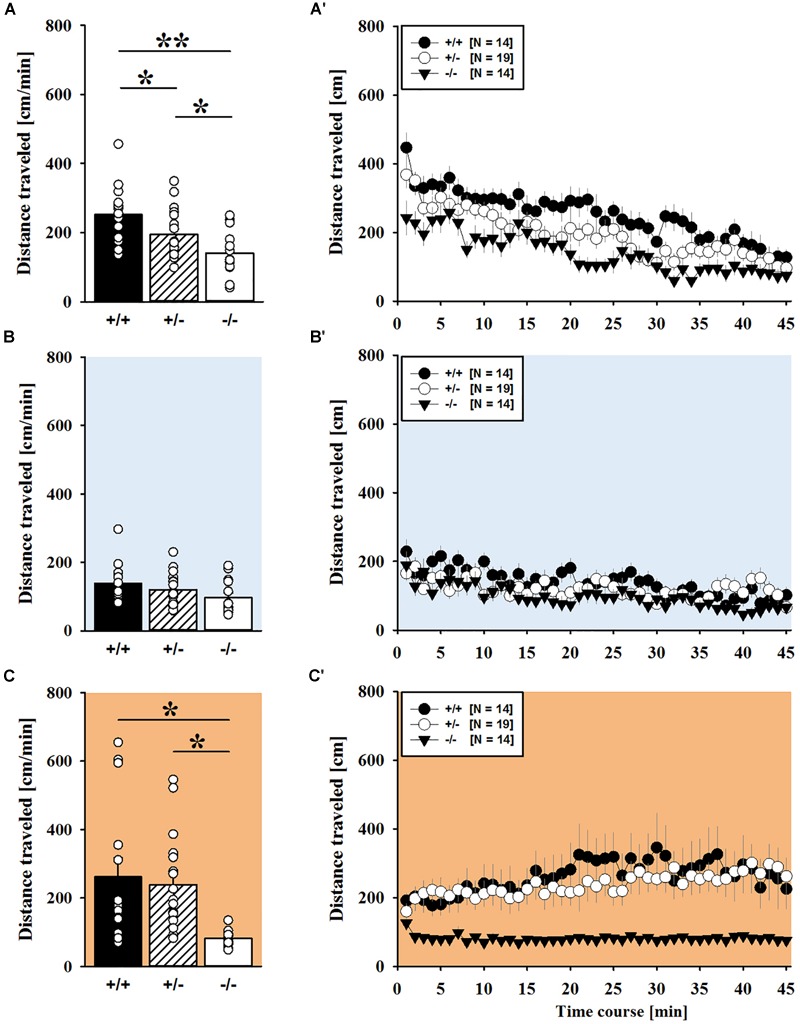
MDMA-induced hyperactivity in adult *Shank1* mice. **(A–C)** Bar graphs and **(A′–C′)** line graphs depicting the distance traveled by *Shank1^+/+^* (black bar/black circle), *Shank1^+/-^* (striped bar/white circle), and *Shank1^-/-^* (white bar/black triangle). Locomotor activity was compared during baseline testing **(A,A′)**, following saline administration **(B,B′)**, and after MDMA treatment **(C,C′)**. Data are presented as means + SEM or means ± SEM. ^∗^*p* < 0.05, ^∗∗^*p* < 0.001.

MDMA administration induced hyperactivity in adult mice (*F*_2,82_ = 9.290, *p* < 0.001), as again evidenced by representative locomotor activity patterns in response to MDMA on test day 3 in comparison to saline on day 2 (Figure 5A). Consistent with AMPH, there was a general genotype effect (*F*_2,41_ = 13.153, *p* < 0.001), with sex having no prominent impact in modulating MDMA effects (*F*_1,41_ = 0.689, *p* = 0.411; sex × genotype: *F*_2,41_ = 4.155, *p* = 0.023; day × sex: *F*_2,82_ = 2.487, *p* = 0.089; day × sex × genotype: *F*_4,82_ = 0.534, *p* = 0.711). Again, this was further supported by a comparison between test days that revealed clearly higher levels of locomotor activity following MDMA treatment than the day before in response to saline (*p* = 0.003; Figure 5B). However, despite this difference held true for *Shank1^+/+^* (*t*_13_ = 2.000, *p* = 0.034, one-tailed) and *Shank1^+/-^* mice (*t*_18_ = 3.356, *p* = 0.004), no evidence for MDMA-induced hyperactivity was evident in *Shank1^-/-^* mice, with their level of locomotor activity being the same following MDMA as in response to saline (*t*_13_ = 1.188, *p* = 0.256; Figure 5B′). In fact, similar to AMPH, although more prominent, MDMA responsivity in adult mice was strongly affected by *Shank1* deletion (day × genotype: *F*_4,82_ = 2.759, *p* = 0.033) and clear genotype differences in hyperactivity following MDMA administration were evident (*F*_2,44_ = 6.606, *p* = 0.003). Importantly, the lack of MDMA-induced hyperactivity in *Shank1^-/-^* mice was not driven by baseline differences in locomotor activity. Although genotype differences were evident under baseline conditions (*F*_2,44_ = 8.286, *p* = 0.001; Figure 6A) and *Shank1^-/-^* mice displayed lower locomotor activity compared to *Shank1^+/+^* (*p* < 0.001) and *Shank1^+/-^* mice (*p* = 0.040), with the latter also differing from each other (*p* = 0.029), locomotor activity did not differ between genotypes in response to saline (*F*_2,44_ = 2.445, *p* = 0.098; Figure 6B). Specifically, as compared to *Shank1^-/-^* mice, increased MDMA-induced hyperactivity was detected in *Shank1^+/+^* (*p* = 0.002) and *Shank1^+/-^* mice (*p* = 0.004), whereas the latter two did not differ from each other (*p* = 0.652). This prominent genotype effect was also reflected in the temporal response pattern (genotype: *F*_2,44_ = 6.606, *p* = 0.003; time × genotype: *F*_88,1936_ = 0.999, *p* = 0.484; Figure 6C). While locomotor activity levels increased in *Shank1^+/+^* and *Shank1^+/-^* mice already from the very beginning of testing, MDMA treatment in *Shank1^-/-^* mice was associated with a decrease of locomotor activity, with very low activity levels being maintained in *Shank1^-/-^* mice throughout testing (Figures 6A′–C′).

Of note, the time spent in the center of the open field was also affected and changed across test days (*F*_2,82_ = 43.972, *p* < 0.001), and genotype-dependent differences were obtained (*F*_2,41_ = 5.374, *p* = 0.008; day × genotype: *F*_4,82_ = 3.264, *p* = 0.016), with sex having no impact (*F*_1,41_ = 0.001, *p* = 0.978; sex × genotype: *F*_2,41_ = 0.823, *p* = 0.446 day × sex: *F*_2,82_ = 0.061, *p* = 0.941; day × sex × genotype: *F*_4,82_ = 0.148, *p* = 0.963). When comparing genotypes on individual test days, genotype differences were evident under baseline conditions (*F*_2,44_ = 5.505, *p* = 0.007; Supplementary Figure 4A). Specifically, *Shank1^+/+^* mice spent more time in the center than *Shank1^+/-^* (*p* = 0.005) and *Shank1^-/-^* mice (*p* = 0.006), with the latter not differing from each other (*p* = 0.883). Genotypes did not differ from each other in terms of the time spent in the center on day 2 in response to saline (*F*_2,44_ = 1.537, *p* = 0.226; Supplementary Figure 4B), and on day 3 in response to MDMA (*F*_2,44_ = 1.170, *p* = 0.320; Supplementary Figure 4C).

Because MDMA-induced hyperactivity was completely absent in *Shank1^-/-^* mice, we also tested the hypothesis that MDMA treatment might have induced repetitive and stereotyped movement patterns, such as circling behavior (Powell et al., 2004; Risbrough et al., 2006). In fact, there was an overall increase in repetitive circling behavior following MDMA treatment (*F*_1,41_ = 78.866, *p* < 0.001). Importantly, however, the increase in circling occurred irrespective of genotype and can thus not explain the lack of MDMA-induced hyperactivity in *Shank1^-/-^* mice. Specifically, there was no prominent effect of genotype (*F*_2,41_ = 1.925, *p* = 0.159; day × genotype: *F*_2,41_ = 1.678, *p* = 0.199) or sex (*F*_1,41_ = 0.004, *p* = 0.953; sex × genotype: *F*_2,41_ = 1.171, *p* = 0.320; day × sex: *F*_1,41_ = 0.061, *p* = 0.806; day × sex × genotype: *F*_2,41_ = 1.695, *p* = 0.196). A comparison between test days revealed that circling behavior following MDMA treatment was clearly higher than the day before in response to saline (*p* < 0.001; Supplementary Figure 5A), yet this increase occurred irrespective of genotype and held true for all individual genotypes (*Shank1^+/+^*: *t*_13_ = 4.283, *p* < 0.001, one-tailed; *Shank1^+/-^*: *t*_18_ = 7.681, *p* < 0.001; *Shank1^-/-^*: *t*_27_ = 4.065, *p* = 0.001; Supplementary Figure 5A′).

### Neurotransmitter Measurements

#### Prefrontal Cortex

**FIGURE 7:**
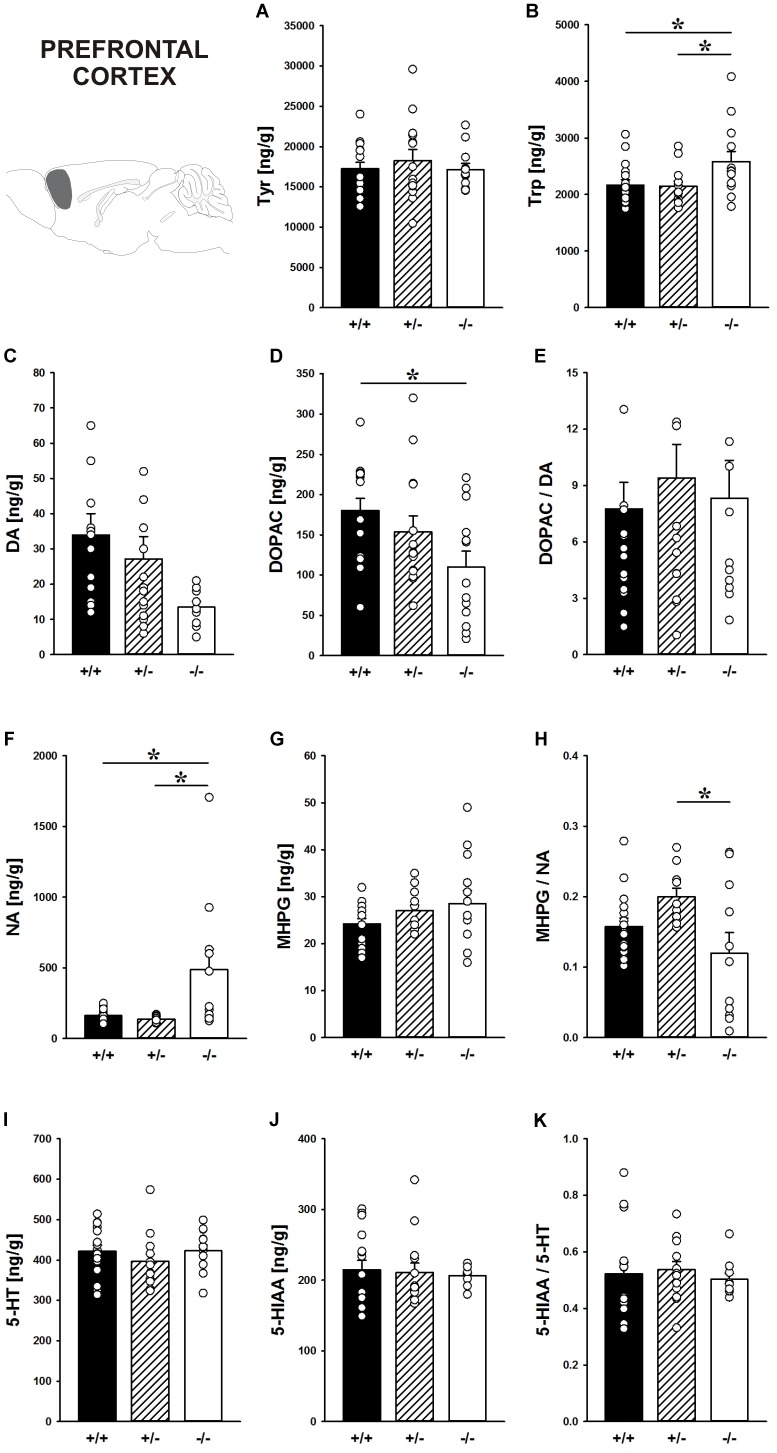
Catecholamine/indolamine, precursor, and metabolite concentrations in the prefrontal cortex of *Shank1* mice. Basal levels of **(A)** Tyrosine (Tyr), **(B)** Tryptophan (Trp), **(C)** Dopamine (DA), **(D)** DA-metabolite dehydroxyphenylacetic acid (DOPAC), **(E)** DOPAC/DA ratio, **(F)** Noradrenaline (NA), **(G)** NA-metabolite 4-hydroxy-3-methoxy-phenylglycol (MHPG), **(H)** MHPG/NA ratio, **(I)** 5-hydroxytryptamine (5-HT; serotonin), **(J)** 5-HT-metabolite 5-hydroxy-indol-acetic acid (5-HIAA), and **(K)** 5-HIAA/5-HT ratio in *Shank1^+/+^* (black bar), *Shank1^+/-^* (striped bar), and *Shank1^-/-^* mice (white bar). *N* = 10–16 per genotype. Data are presented as means + SEM. ^∗^*p* < 0.05. Schematic representation of the prefrontal cortex was adapted from Paxinos and Franklin (2001).

Concentrations of the DA precursor Tyr in the prefrontal cortex did not differ between genotypes (*F*_2,39_ = 0.364, *p* = 0.697; Figure 7A). *Shank1* deletion, however, affected the level of the 5-HT precursor Trp (*F*_2,40_ = 3.933, *p* = 0.028; Figure 7B). Specifically, *Shank1^-/-^* mice had higher Trp concentrations in the prefrontal cortex than *Shank1^+/+^* (*p* = 0.019) and *Shank1^+/-^* mice (*p* = 0.018), with the latter two not differing from each other (*p* = 0.910).

Although basal DA levels clearly tended to be lower in *Shank1^-/-^* mice, apparent genotype differences in DA levels did not reach statistical significance (*F*_2,38_ = 3.192, *p* = 0.052; Figure 7C), while the concentration of the DA metabolite DOPAC differed between genotypes (*F*_2,40_ = 3.879, *p* = 0.029; Figure 7D). *Shank1^+/+^* mice had higher DOPAC concentrations than *Shank1^-/-^* (*p* = 0.008) but not *Shank1^+/-^* mice (*p* = 0.290), with *Shank1^-/-^* and *Shank1^+/-^* mice also not differing from each other (*p* = 0.102). The DOPAC/DA ratio, however, was not affected by *Shank1* deletion (*F*_2,38_ = 0.261, *p* = 0.772; Figure 7E).

While DA levels were not significantly affected by genotype, *Shank1* deletion affected basal NA concentrations (*F*_2,35_ = 6.597, *p* = 0.004; Figure 7F). *Shank1^-/-^* mice had higher concentrations of NA as compared to *Shank1^+/+^* (*p* = 0.003) and *Shank1^+/-^* mice (*p* = 0.003), while *Shank1^+/+^* and *Shank1^+/-^* mice did not differ in that aspect (*p* = 0.797). There were no differences between genotypes in the concentrations of the NA metabolite MHPG (*F*_2,39_ = 1.604, *p* = 0.214; Figure 7G), yet the MHPG/NA ratio differed between genotypes (*F*_2,34_ = 4.066, *p* = 0.026; Figure 7H). The MHPG/NA ratio was lower in *Shank1^-/-^* mice as compared to *Shank1^+/-^* mice (*p* = 0.007) but not as compared to *Shank1^+/+^* mice (*p* = 0.140). No significant differences in MHPG/NA ratio between *Shank1^+/+^* and *Shank1^+/-^* mice were detected (*p* = 0.114).

The 5-HT system was not affected by *Shank1* deletion. Specifically, basal 5-HT concentrations, the levels of the main 5-HT metabolite 5-HIAA, and the 5-HIAA/5-HT ratio did not differ between genotypes (all *p*-values > 0.100; Figures 7I–K).

#### Nucleus Accumbens

**FIGURE 8:**
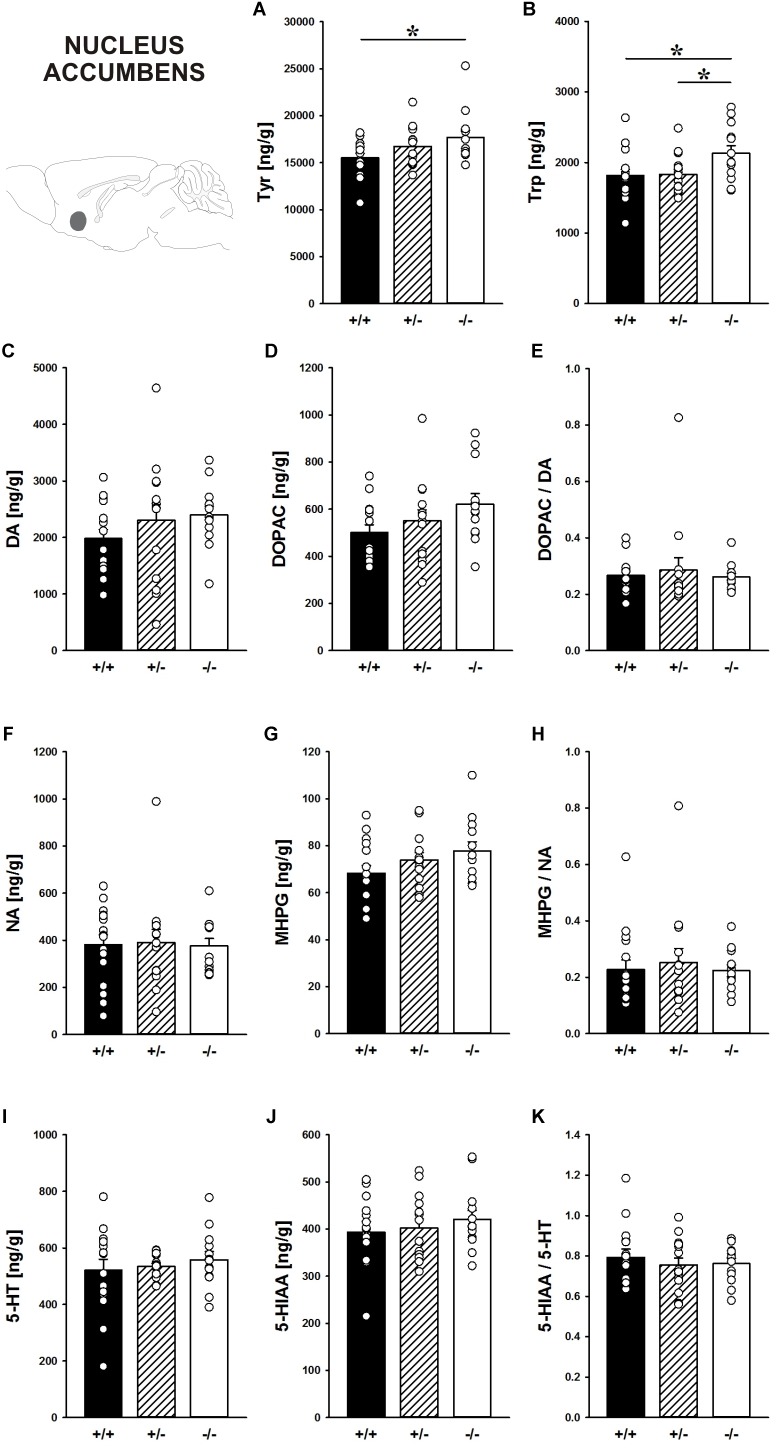
Catecholamine/indolamine, precursor, and metabolite concentrations in the nucleus accumbens of *Shank1* mice. Basal levels of **(A)** Tyrosine (Tyr), **(B)** Tryptophan (Trp), **(C)** Dopamine (DA), **(D)** DA-metabolite dehydroxyphenylacetic acid (DOPAC), **(E)** DOPAC/DA ratio, **(F)** Noradrenaline (NA), **(G)** NA-metabolite 4-hydroxy-3-methoxy-phenylglycol (MHPG), **(H)** MHPG/NA ratio, **(I)** 5-hydroxytryptamine (5-HT; serotonin), **(J)** 5-HT-metabolite 5-hydroxy-indol-acetic acid (5-HIAA), and **(K)** 5-HIAA/5-HT ratio in *Shank1^+/+^* (black bar), *Shank1^+/-^* (striped bar), and *Shank1^-/-^* mice (white bar). *N* = 13–16 per genotype. Data are presented as means + SEM. ^∗^*p* < 0.05. Schematic representation of the nucleus accumbens was adapted from Paxinos and Franklin (2001).

In the nucleus accumbens, *Shank1* deletion affected the concentration of the DA precursor Tyr (*F*_2,40_ = 3.421, *p* = 0.043; Figure 8A) and the 5-HT precursor Trp (*F*_2,40_ = 3.709, *p* = 0.033; Figure 8B). Specifically, *Shank1^-/-^* mice had higher Tyr concentrations than *Shank1^+/+^* mice (*p* = 0.013) but not *Shank1^+/-^* mice (*p* = 0.281), with the latter also not differing from each other (*p* = 0.143). Trp levels in *Shank1^-/-^* mice were higher than in *Shank1^+/+^* (*p* = 0.018) and *Shank1^+/-^* mice (*p* = 0.028), whereas the latter did not differ from each other (*p* = 0.901).

However, despite genotype differences in the levels of the precursors, there were no differences between genotypes in DA, DOPAC, DOPAC/DA ratio, NA, MHPG, MHPG/NA ratio, 5-HT, 5-HIAA, and 5-HIAA/5-HT ratio (all *p*-values > 0.100; Figures 8C–K).

#### Hypothalamus

**FIGURE 9:**
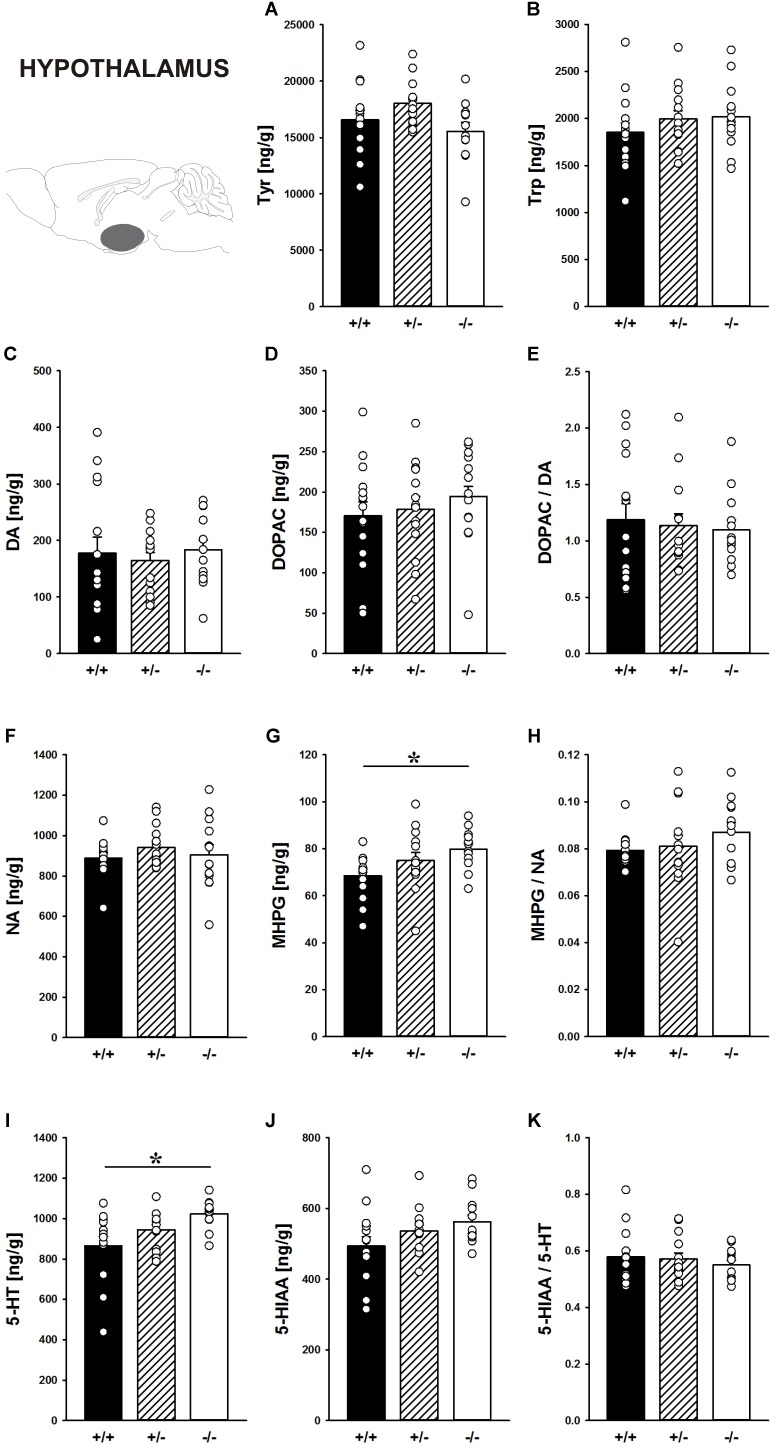
Catecholamine/indolamine, precursor, and metabolite concentrations in the hypothalamus of *Shank1* mice. Basal levels of **(A)** Tyrosine (Tyr), **(B)** Tryptophan (Trp), **(C)** Dopamine (DA), **(D)** DA-metabolite dehydroxyphenylacetic acid (DOPAC), **(E)** DOPAC/DA ratio, **(F)** Noradrenaline (NA), **(G)** NA-metabolite 4-hydroxy-3-methoxy-phenylglycol (MHPG), **(H)** MHPG/NA ratio, **(I)** 5-hydroxytryptamine (5-HT; serotonin), **(J)** 5-HT-metabolite 5-hydroxy-indol-acetic acid (5-HIAA), and **(K)** 5-HIAA/5-HT ratio in *Shank1^+/+^* (black bar), *Shank1^+/-^* (striped bar), and *Shank1^-/-^* mice (white bar). *N* = 12–15 per genotype. Data are presented as means + SEM. ^∗^*p* < 0.05. Schematic representation of the hypothalamus was adapted from Paxinos and Franklin (2001).

Concentrations of the precursors Tyr tended to be affected by *Shank1* deletion in the hypothalamus (*F*_2,38_ = 2.902, *p* = 0.067; Figure 9A). Trp in the hypothalamus did not differ between genotypes (*F*_2,39_ = 0.864, *p* = 0.429; Figure 9B).

Moreover, the DA system was not affected by *Shank1* deletion. DA, DOPAC, and DOPAC/DA ratio did not differ between genotypes (all *p*-values > 0.100; Figures 9C–E).

While NA concentrations were not affected by *Shank1* deletion (*F*_2,37_ = 0.598, *p* = 0.555; Figure 9F), MHPG levels differed between genotypes (*F*_2,37_ = 3.596, *p* = 0.037; Figure 9G). Specifically, MHPG concentrations in the hypothalamus of *Shank1^-/-^* mice were higher than *Shank1^+/+^* mice (*p* = 0.012) but not *Shank1^+/-^* mice (*p* = 0.263), with no difference between *Shank1^+/+^* and *Shank1^+/-^* mice (*p* = 0.121). Genotype-dependent differences in the MHPG/NA ratio were not evident (*F*_2,36_ = 1.041, *p* = 0.363; Figure 9H).

Finally, there were genotype-dependent differences in 5-HT concentrations in the hypothalamus (*F*_2,38_ = 5.437, *p* = 0.008; Figure 9I). While *Shank1^-/-^* mice had higher 5-HT concentrations than *Shank1^+/+^* mice (*p* = 0.002), this difference was not present between *Shank1^-/-^* and *Shank1^+/-^* mice (*p* = 0.113) or between *Shank1^+/+^* and *Shank1^+/-^* mice (*p* = 0.095). In conjunction, there was a trend for differences in 5-HIAA concentrations (*F*_2,38_ = 2.581, *p* = 0.089; Figure 9J). No genotype-dependent differences in the 5-HIAA/5-HT ratio were observed (*F*_2,38_ = 0.480, *p* = 0.622; Figure 9K).

## Discussion

The goal of our present study was to test whether *Shank1* plays a role in the behavioral effects of psychostimulants and whether this is associated with genotype-dependent neurochemical alterations. To this aim, null mutant *Shank1^-/-^* mice were treated with AMPH (2.5 mg/kg) and MDMA (20 mg/kg), and psychostimulant-induced hyperactivity was compared to heterozygous *Shank1^+/-^* and wildtype *Shank1^+/+^* littermate controls. Results show that *Shank1^-/-^* mice display reduced psychostimulant-induced hyperactivity, although psychostimulants robustly stimulated locomotor activity in littermate controls. *Shank1* deletion effects emerged throughout development, were particularly prominent in adulthood, and seen in response to both psychostimulants, i.e., AMPH and MDMA.

Mutations in *SHANK* genes are associated with multiple major neuropsychiatric disorders, including SCZ and BPD besides ASD (Guilmatre et al., 2014; Bourgeron, 2015; de la Torre-Ubieta et al., 2016). Because psychostimulants, such as AMPH, can provoke mania-like symptoms in healthy subjects and exacerbate symptoms or induce a manic episode in BPD patients (Meyendorff et al., 1985; Peet and Peters, 1995; Hasler et al., 2006), we expected that *Shank1* deletion leads to increased AMPH-induced hyperactivity. In fact, AMPH-induced hyperactivity is a commonly applied paradigm to assess behavioral phenotypes related to BPD and considered to be the gold standard for modeling mania-like elevated drive in mouse models (Berggren et al., 1978; Gould et al., 2001; Kato et al., 2007; Young et al., 2011). Unexpectedly, however, *Shank1^-/-^* mice displayed reduced psychostimulant-induced hyperactivity. This was reflected in a weaker increase in locomotor activity and a complete lack of induction of rearing behavior following AMPH. Supporting a gene dosage effect, *Shank^+/-^* mice displayed an intermediate phenotype. While locomotor activity was increased following AMPH to levels similar to littermate controls, induction of rearing behavior following AMPH was absent, with rearing levels being similar to *Shank1^-/-^* mice. Our observation that AMPH-induced hyperactivity is reduced rather than enhanced following *Shank1* deletion thus clearly speaks against a behavioral phenotype with relevance to BPD. The lack of a BPD-like phenotype is consistent with currently available human data linking mutations in *SHANK2* and *SHANK3* but not *SHANK1* to BPD. Specifically, a duplication in *SHANK2* has been reported for a BPD patient (Noor et al., 2014) and four point mutations in *SHANK2* (c.3979G > A; c.2900A > G; c.4461C > T; c.4926G > A) have been identified very recently in BPD patients (Yang et al., 2018). Moreover, in a substantial number of individuals lacking *SHANK3* and diagnosed with the Phelan-McDermid 22q13 deletion syndrome BPD was evident (Sovner et al., 1996; Willemsen et al., 2011; Denayer et al., 2012; Verhoeven et al., 2012, 2013; Vucurovic et al., 2012). Notably, *SHANK3* duplications were also associated with BPD in humans (Han et al., 2013). In contrast, mutations in *SHANK1* have been associated with SCZ but not BPD. Fromer et al. (2014) reported a *de novo SHANK1* frameshift mutation in a SCZ patient. Lennertz et al. (2012) found that the T allele of the *SHANK1* promotor variant rs3810280 leads to impaired auditory working memory capacity as assessed with digit span in SCZ patients and subjects clinically at risk for developing a psychosis but not in healthy controls.

The clinical pattern is reflected in relevant *Shank* mouse models. For instance, Pappas et al. (2017) recently reported BPD-like phenotypes in *Shank2^Δ24-/-^* mice. Specifically, they observed increased locomotor activity indicative of elevated drive and perturbed circadian rhythms, which could be normalized by lithium and valproate treatment. Importantly, the increase in locomotor activity caused by *Shank2* deletion could be augmented by AMPH administration and the augmentation was more prominent in *Shank2^Δ24-/-^* mice than in *Shank2^Δ24+/+^* controls (Pappas et al., 2017). *Shank2* deficiency limited to the forebrain recapitulated the BPD-like phenotype, which was associated with alterations in the composition and function of NMDA and AMPA receptors (Pappas et al., 2017). Moreover, *Shank2^Δ7-/-^* mice likewise display enhanced levels of locomotor activity but augmentation by methylphenidate was weaker in *Shank2^Δ7-/-^* mice than in *Shank2^Δ7+/+^* controls (Ey et al., 2018). Finally, *Shank3* overexpression in mice was linked to a BPD-like phenotype characterized by increased locomotor activity and abnormal circadian rhythms (Han et al., 2013). Importantly, hyperactivity of *Shank3* overexpressing mice following an acute injection of AMPH aggravated to a greater extent than in controls. The BPD-like phenotype was rescued by valproate but not lithium treatment. To our knowledge, no studies on *Shank3* deletion effects on AMPH-induced hyperactivity are available. Together, this might suggest that *Shank1* deletion leads to specific alterations in brain structure or function reducing rather than enhancing the efficacy of AMPH to stimulate hyperactivity not present in other relevant *Shank* mouse models.

Sold as speed, AMPH is among the most commonly used illicit drugs, but is also available by prescription and is widely used for treating weight control, narcolepsy, and attention deficit disorder (Sulzer et al., 2005). AMPH acts primarily as a DA and NA releaser by induction of reverse transport of catecholamines through plasma membrane uptake carries, i.e., the DA and NA transporters, and redistribution of catecholamines from synaptic vesicles to the cytosol, with little affinity for receptors (Sulzer et al., 2005; Hutson et al., 2014), although prominent effects on the 5-HT system were also reported (Pum et al., 2007). In mice, the stimulatory effects of AMPH are widely believed to be driven by increased synaptic DA levels and subsequent activation of postsynaptic receptors. In fact, while mice deficient for the NA transporter are supersensitive (Xu F. et al., 2000), AMPH-induced hyperactivity is enhanced in DA transporter overexpressing mice (Salahpour et al., 2008) but completely abolished in mice lacking the DA transporter (Giros et al., 1996), an effect mediated by the 5-HT system (Gainetdinov et al., 1999). Yet, it is not affected by deletion of the gene for the 5-HT transporter (Bengel et al., 1998), although, to a lesser extent, AMPH affects 5-HT transporters as well (Steele et al., 1987; Crespi et al., 1997). Postsynaptically, AMPH action was reported to depend on D2 but less on D3 and not on D4 receptors, with mixed results for the D1 receptor (Xu M. et al., 2000; McNamara et al., 2006; Fan and Hess, 2007; Fan et al., 2010). Various 5-HT receptors play a modulating role as well and it was shown that AMPH-induced hyperactivity is increased in 5-HT1A (van den Buuse et al., 2011), 5-HT1B (Bronsert et al., 2001), and 5-HT2A (Salomon et al., 2007) receptor knockout mice. Partially conflicting evidence was obtained in pharmacological studies, with 8-OH-DPAT (Przegalinski et al., 2000) and SB216641 (Przegalinski et al., 2001), 5-HT1A agonist and 5-HT1B antagonist, respectively, reducing AMPH-induced hyperactivity, while CP94,253, a 5-HT1B agonist, further enhances AMPH-induced hyperactivity (Przegalinski et al., 2001). Mixed results were obtained for the 5-HT2A (Carlsson et al., 1999; Sorensen et al., 1993) and the 5-HT2C (O’Neill et al., 1999; Marquis et al., 2007) receptor. For a more comprehensive overview including other species besides mice, the reader is referred to two excellent reviews (Hutson et al., 2014; Müller and Homberg, 2015).

While AMPH-induced hyperactivity was reduced but still detectable in *Shank1^-/-^* mice, MDMA-induced hyperactivity was robustly blocked and completely absent in *Shank1^-/-^* mice. Because a genetic manipulation affecting psychostimulant-induced hyperactivity may easily look like an attenuation when the dose-response curve is in fact shifted to the left, but now producing motor stereotypies instead of ambulation, we measured repetitive circling behavior (Powell et al., 2004; Risbrough et al., 2006). We focused on mice treated with MDMA, mostly for two reasons. Firstly, the *Shank1* deletion effect was most prominent in response to MDMA. Secondly, the MDMA dose applied here can be considered relatively high, making it particularly likely that motor stereotypies, such as repetitive circling behavior occur. However, while there was an overall increase in repetitive circling behavior following MDMA treatment, the increase in circling occurred irrespective of genotype and can thus not explain the lack of MDMA-induced hyperactivity in *Shank1^-/-^* mice. Although this speaks against a dose-response curve that is shifted to the left following *Shank1* deletion, a future dose-response study including several MDMA doses appears warranted.

Sold as ecstasy, MDMA is also among the most widely used illicit drugs, yet with little therapeutic use (Sulzer et al., 2005). MDMA acts primarily as a 5-HT and DA releaser by blocking reuptake transporters. However, relative to AMPH, MDMA is more potent at releasing 5-HT than DA (Steele et al., 1987; Crespi et al., 1997). In mice, it was repeatedly shown that MDMA leads to hyperactivity, and hyperactivity induced by low doses of MDMA of up to 10 mg/kg has previously been found to be blocked by deletion of the genes for either the 5-HT1B receptor (Scearce-Levie et al., 1999; Jean et al., 2012), the 5-HT2B receptor (Doly et al., 2008; Doly et al., 2009), or the 5-HT transporter (Bengel et al., 1998), while it is potentiated in 5-HT2A receptor knockout mice (Orejarena et al., 2011). Consistently, MDMA-induced hyperactivity was reported to be reduced by the 5-HT1B antagonist GR127935 (Scearce-Levie et al., 1999), the 5-HT2B antagonist RS127445 (Doly et al., 2008; Doly et al., 2009), the 5-HT2C antagonist RS102221 (Conductier et al., 2005), the 5-HT4 antagonist RS39604 (Jean et al., 2012), and the 5-HT uptake inhibitor fluoxetine (Fantegrossi et al., 2003). Higher MDMA doses, however, induced late-phase increases in hyperactivity in 5-HT1B (Scearce-Levie et al., 1999) and 5-HT2B (Doly et al., 2009) receptor knockout mice, possibly due to a delayed increase in DA release (White et al., 1994, 1996; Yamamoto and Spanos, 1988). Risbrough et al. (2006) found that late-phase increases in hyperactivity evoked by MDMA are enhanced in D1 knockout mice but reduced in D2 and D3 knockout mice. In DA transporter knockout mice, the typical hyperactivity displayed by these mice is reduced in response to MDMA (Powell et al., 2004). Benturquia et al. (2008) reported that MDMA-induced hyperactivity is antagonized by SCH23390, a D1 antagonist. Alike for AMPH, the reader is referred to two excellent reviews for a more comprehensive overview including other species besides mice (Sulzer et al., 2005; Müller and Homberg, 2015).

Very little is known about interactions between SHANK1 and the major neurotransmitters affected by AMPH and MDMA, i.e., DA, NA, and 5-HT. Azdad et al. (2009) obtained evidence indicating that in striatal neurons D2 receptors regulate NMDA-mediated neuronal excitation resulting in a depolarized plateau potential and spike firing through a mechanism requiring scaffolding proteins of the SHANK family including SHANK1. Buonaguro et al. (2017) showed that 3 weeks of treatment with the D2 antagonist haloperidol led to a reduction of SHANK1 mRNA expression in the anterior cingulate cortex and the insula as well as the nucleus accumbens. Li et al. (2015) reported that vortioxetine but not fluoxetine treatment leads to enhanced levels of SHANK1 mRNA in the hippocampus. Vortioxetine acts through increasing 5-HT concentrations in the synaptic cleft by inhibiting reuptake and through modulating 5-HT receptors. Finally, Pal and Das (2013) reported upregulation of SHANK1 mRNA and protein levels in cortex and midbrain following chronic morphine treatment.

In an initial effort to better understand the effects of *Shank1* deletion on the neurochemical architecture, we analyzed DA, NA, and 5-HT neurotransmitter concentrations together with their precursors and metabolites in relevant brain structures, namely prefrontal cortex, nucleus accumbens, and hypothalamus. In the prefrontal cortex, which plays a key role in maintaining inhibitory control over striatal mechanisms involved in the effects of drugs of abuse (Everitt and Robbins, 2016), basal DA levels tended to be affected by *Shank1* deletion, with lower DA concentrations in *Shank1^-/-^* mice. Moreover, the concentration of the DA metabolite DOPAC differed between genotypes and DOPAC levels were reduced in *Shank1^-/-^* mice. The DOPAC/DA ratio, however, was not affected. Concentrations of the DA precursor Tyr also did not differ between genotypes. Besides DA, basal NA concentrations in the prefrontal cortex were affected by *Shank1* deletion, with higher NA concentrations in *Shank1^-/-^* mice. There were no differences between genotypes in the concentrations of the NA metabolite MHPG, yet the MHPG/NA ratio differed between genotypes and was lower in *Shank1^-/-^* mice. Importantly, the decreased MHPG/NA ratio in *Shank1^-/-^* mice, which was paralleled by increases in NA concentrations in absence of changes in MHPG, might indicate decreased NA metabolism in *Shank1^-/-^* mice. Finally, *Shank1* deletion affected the level of the 5-HT precursor Trp in the prefrontal cortex, with *Shank1^-/-^* mice having higher Trp concentrations. However, basal 5-HT concentrations, the levels of the main 5-HT metabolite 5-HIAA, and the 5-HIAA/5-HT ratio did not differ between genotypes.

In the nucleus accumbens, a key brain structure strongly implicated in the rewarding actions of drugs of abuse (Everitt and Robbins, 2016), *Shank1* deletion affected the concentration of the DA precursor Tyr and the 5-HT precursor Trp, with the levels of both precursors being higher in *Shank1^-/-^* mice. Despite these genotype differences in the levels of the precursors, DA, NA, and 5-HT concentrations as well as their metabolites were not affected in this brain region.

In the hypothalamus, a brain region less heavily involved in the actions of drugs of abuse (Everitt and Robbins, 2016), no prominent genotype effects on the levels of the precursors Tyr and Trp were evident and DA, DOPAC as well as the DOPAC/DA ratio also did not differ between genotypes. However, while NA concentrations were not affected by *Shank1* deletion, MHPG levels were higher in the hypothalamus of *Shank1^-/-^* mice. Genotype-dependent differences in the MHPG/NA ratio were not evident. Finally, there were genotype-dependent differences in 5-HT concentrations in the hypothalamus, with *Shank1^-/-^* mice having higher concentrations. In conjunction, there was a trend for a similar genotype pattern in 5-HIAA concentrations. No genotype-dependent differences in the 5-HIAA/5-HT ratio were observed.

Together, this shows that *Shank1* deletion leads to alterations in the neurochemical architecture of the brain regions evaluated, with most prominent effects being evident in the prefrontal cortex. This is remarkable because SHANK1 expression is particularly high in cortex, but low in striatum and hypothalamus (Peça et al., 2011), suggesting local effects of *Shank1* deletion. It is tempting to speculate that the opposite alterations in DA and NA concentrations together with the decreased NA metabolism in the prefrontal cortex of *Shank1^-/-^* mice affect the fronto-striatal circuitry in mediating inhibitory control. Reduced prefrontal inhibitory control was strongly implicated in dominance of subcortically mediated responding to drugs of abuse (Everitt and Robbins, 2016). Of note, the increase of Trp in prefrontal cortex and nucleus accumbens is interesting because the essential amino acid Trp is not only the precursor of 5-HT, but also gives rise to melatonin and kynurenines, which have all been implicated, in one or the other way, in ASD, SCZ, and BPD (Morera-Fumero and Abreu-Gonzalez, 2013; Rossignol and Frye, 2014; Geoffroy et al., 2015; Lim et al., 2016; Erhardt et al., 2017).

To our knowledge, it is currently not known whether *Shank1* deletion affects the expression and/or function of the AMPH and MDMA targets, i.e., plasma membrane uptake carries for DA, NA, and 5-HT. Because reduced efficacy to stimulate hyperactivity in *Shank1^-/-^* mice is seen in response to both AMPH and MDMA, alterations in the NA transporter are not prime candidates driving the effects. Another candidate for the reduced efficacy of psychostimulants to stimulate hyperactivity in *Shank1^-/-^* mice is the DA transporter. Although reduced expression and/or function of the DA transporter could explain reduced efficacy of psychostimulants, this also appears unlikely to be the case. This is because DA transporter knockout mice display increased locomotor activity under drug free conditions (Giros et al., 1996; Gainetdinov et al., 1999; Powell et al., 2004), whereas *Shank1* deletion leads to mild hypoactivity (Hung et al., 2008; Silverman et al., 2011; Wöhr et al., 2011). Finally, the 5-HT transporter appears of relevance because MDMA-induced hyperactivity is abolished in mice lacking the 5-HT transporter (Bengel et al., 1998), yet this as well does not explain the full result pattern because AMPH-induced hyperactivity is not affected by deletion of the gene for the 5-HT transporter (Bengel et al., 1998). Of note, the time spent in the center was not affected by AMPH or MDMA treatment in a genotype-dependent manner and the reduction in center time seen following *Shank1* deletion might reflect a mild increase in anxiety levels and is consistent with the literature (Hung et al., 2008; Silverman et al., 2011). Together, this might thus indicate that postsynaptic receptors implicated in psychostimulant-induced hyperactivity, such as D1 and D2 (Xu M. et al., 2000; Fan and Hess, 2007; Fan et al., 2010) as well as 5-HT1B (Scearce-Levie et al., 1999; Jean et al., 2012) and 5-HT2B (Doly et al., 2008; Doly et al., 2009) receptors, drive the effects of *Shank1* deletion. However, to our knowledge, there is no study on the expression of relevant receptors in mice lacking SHANK1 available. Future studies on the effects of *Shank1* deletion on DA, NA, and 5-HT transporters and receptors appear therefore warranted.

As master scaffolding proteins enriched in the PSD at excitatory glutamatergic synapses, SHANKs anchor glutamate receptors and link them to the actin cytoskeleton and postsynaptic signaling pathways (Ting et al., 2012; Sala et al., 2015). In the first study on the effects of *Shank1* deletion, Hung et al. (2008) focused on the forebrain and hippocampus and demonstrated that *Shank1* is important for regulating dendritic spine morphology and synaptic strength. Standard measures of synaptic plasticity, however, were unchanged, with intact hippocampal long-term potentiation. NMDA, AMPA, and metabotropic glutamate receptors were also not affected (Hung et al., 2008). AMPA and NMDA receptors appear to play a role in acquisition and reinstatement of conditioned place preference, as, for instance, induced by MDMA (García-Pardo et al., 2015; García-Pardo et al., 2018).

More recently, Sungur et al. (2017) studied the effects of *Shank1* deletion on protein expression levels of the brain-derived neurotrophic factor BDNF together with its epigenetic regulation. Partial genetic depletion of BDNF was repeatedly associated with stronger AMPH-induced hyperactivity and associated with increased striatal DA concentrations (Dluzen et al., 2001; Saylor and McGinty, 2008; Manning et al., 2016). Similar findings were obtained for MDMA (Mouri et al., 2017). Sungur et al. (2017) found that *Shank1* deletion has no effect on basal BDNF expression levels, yet in response to learning a memory task hippocampal BDNF expression was particularly enhanced in *Shank1^-/-^* mice. A subsequent investigation of the epigenetic regulation revealed enrichment of histone H3 acetylation at the *Bdnf* promoter1 in *Shank1^-/-^* mice. Because BDNF is an important regulator of gene expression stimulating D3 receptor expression (Guillin et al., 2001) and D3 receptor activation was reported to inhibit psychostimulant-induced hyperactivity (McNamara et al., 2006), altered BDNF expression and its epigenetic regulation might thus be associated with the reduced efficacy of psychostimulants to stimulate hyperactivity in *Shank1^-/-^* mice.

Finally, another relevant factor that possibly contributes to the reduced efficacy of psychostimulants in *Shank1^-/-^* mice might be parvalbumin (PV) expression. PV is a calcium-binding protein important for the maintenance of the excitation/inhibition balance in the brain (Hu et al., 2014), repeatedly associated with major neuropsychiatric disorders, including ASD, SCZ, and BPD (Marín, 2012). Acute AMPH treatment stimulates robust firing of striatal PV-positive GABAergic interneurons (Wiltschko et al., 2010). Moreover, AMPH was reported to induce expression of the activity-inducible transcription factor Fos in PV-positive GABAergic interneurons in the nucleus accumbens, together with phosphorylation of the methyl-DNA-binding protein MeCP2 at Ser421 (Deng et al., 2010), the latter being associated with SHANK1 protein expression (Du et al., 2016). Very recently, it was further shown that AMPH treatment in mice with silenced PV-positive GABAergic interneurons evoked stronger activation in both D1 and D2 receptor-expressing medium spiny neurons in the nucleus accumbens (Wang et al., 2018). Importantly, silencing PV-positive GABAergic interneurons in the nucleus accumbens selectively inhibited the expression of locomotor sensitization following repeated injections of AMPH and blocked AMPH-induced conditioned place preference without affecting AMPH-induced DA release and hyperactivity (Wang et al., 2018). This is relevant because SHANK1 protein is highly co-localized with PV-expressing fast-spiking inhibitory interneurons (Mao et al., 2015) and *Shank1* deletion was shown to result in reduced PV expression (Filice et al., 2016). It would thus be interesting to test whether psychostimulant-induced hyperactivity is altered in PV-deficient mice, which display behavioral phenotypes with relevance to ASD (Wöhr et al., 2015). Specifically, Filice et al. (2016) found that the reduction of PV-immunoreactive neurons caused by *Shank1* deletion was due to a reduction in *Pvalb* mRNA and PV protein, without any indication for PV-expressing GABAergic interneuron loss. Importantly, PV protein expression levels were selectively decreased in those brain regions normally expressing high levels of SHANK1, such as the somatosensory cortex. However, no evidence for effects of *Shank1* deletion was obtained in the striatum, a region with low SHANK1 expression levels in *Shank1^+/+^* mice, similar to the local effects on neurochemical architecture in the present study.

## Conclusion

*Shank1^-/-^* mice display reduced psychostimulant-induced hyperactivity, although psychostimulants robustly stimulated locomotor activity in littermate controls. *Shank1* deletion effects emerged throughout development, were particularly prominent in adulthood, and seen in response to both psychostimulants, i.e., AMPH and MDMA. Specifically, while AMPH-induced hyperactivity was reduced but still detectable in *Shank1^-/-^* mice, MDMA-induced hyperactivity was robustly blocked and completely absent in *Shank1^-/-^* mice. Reduced efficacy of psychostimulants to stimulate hyperactivity in *Shank1^-/-^* mice might be associated with alterations in the neurochemical architecture in prefrontal cortex, nucleus accumbens, and hypothalamus. Our observation that psychostimulant-induced hyperactivity is reduced rather than enhanced in *Shank1^-/-^* mice clearly speaks against a behavioral phenotype with relevance to BPD. Lack of BPD-like phenotype is consistent with currently available human data linking mutations in *SHANK2* and *SHANK3* but not *SHANK1* to BPD.

## Author Contributions

AS, TR, EA, and WD performed the experiments and/or data analysis. AS, RS, AdR, and MW wrote the manuscript. AS, AdR, and MW designed the study and supervised the project. MW acquired funding. All authors were involved in data interpretation.

## Conflict of Interest Statement

The authors declare that the research was conducted in the absence of any commercial or financial relationships that could be construed as a potential conflict of interest.

## Supplementary Material

FIGURE S1 Time spent in the center in juvenile *Shank1* mice treated with AMPH. **(A–C)** Bar graphs depicting the time spent in the center by *Shank1^+/+^* (black bar), *Shank1^+/-^* (striped bar), and *Shank1^-/-^* (white bar). Center time was compared during baseline testing **(A)**, following saline administration **(B)**, and after AMPH treatment **(C)**. Data are presented as means + SEM. ^∗^*p* < 0.05, ^∗∗^*p* < 0.001.

FIGURE S2 AMPH-induced rearing behavior in adult *Shank1* mice. **(A–C)** Bar graphs and **(A′–C′)** line graphs depicting the rearing behavior by *Shank1^+/+^* (black bar/black circle), *Shank1^+/-^* (striped bar/white circle), and *Shank1^-/-^* (white bar/black triangle). Rearing behavior was compared during baseline testing **(A,A′)**, following saline administration **(B,B′)**, and after AMPH treatment **(C,C′)**. Data are presented as means + SEM or means ± SEM. ^∗∗^*p* < 0.001.

FIGURE S3 Time spent in the center in adult *Shank1* mice treated with AMPH. **(A–C)** Bar graphs depicting the time spent in the center by *Shank1^+/+^* (black bar), *Shank1^+/-^* (striped bar), and *Shank1^-/-^* (white bar). Center time was compared during baseline testing **(A)**, following saline administration **(B)**, and after AMPH treatment **(C)**. Data are presented as means + SEM. ^∗^*p* < 0.05.

FIGURE S4 Time spent in the center in adult *Shank1* mice treated with MDMA. **(A–C)** Bar graphs depicting the time spent in the center by *Shank1^+/+^* (black bar), *Shank1^+/-^* (striped bar), and *Shank1^-/-^* (white bar). Center time was compared during baseline testing **(A)**, following saline administration **(B)**, and after MDMA treatment **(C)**. Data are presented as means + SEM. ^∗^*p* < 0.05.

FIGURE S5 MDMA-induced repetitive and stereotyped circling behavior in adult *Shank1* mice treated with MDMA. **(A)** Bar graph depicting the circling behavior displayed by all genotypes following saline administration (blue bar) and after MDMA treatment (orange bar). **(A′)** Line graph depicting the circling behavior displayed by *Shank1^+/+^* (black circle), *Shank1^+/-^* (white circle), and *Shank1^-/-^* (black triangle) over two consecutive test days, i.e., following saline administration (blue are) and after MDMA treatment (orange area). Data are presented as means + SEM or means ± SEM. ^##^*p* < 0.001 **(A)**, ^#^*p* < 0.05 and ^##^*p* < 0.001 vs. day 2 **(A′)**.
