# Supplementary figures and images for: Reduced Efficacy of d-Amphetamine and 3,4-Methylenedioxymethamphetamine in Inducing Hyperactivity in Mice Lacking the Postsynaptic Scaffolding Protein SHANK1
Source: Front Mol Neurosci. 2018 Nov 16;11:419. doi: 10.3389/fnmol.2018.00419 (PMC6250831; doi:10.3389/fnmol.2018.00419)

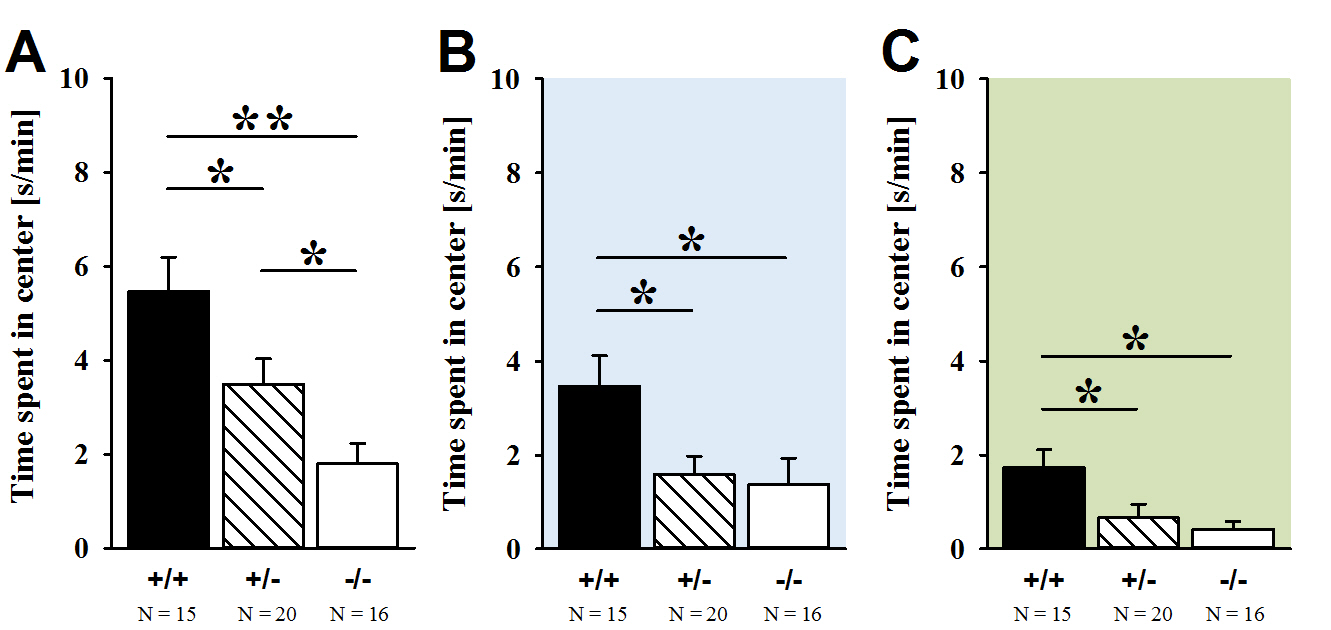

Supplement: FIGURE S1 — Time spent in the center in juvenile Shank1 mice treated with AMPH. (A–C) Bar graphs depicting the time spent in the center by Shank1+/+ (black bar), Shank1+/- (striped bar), and Shank1-/- (white bar). Center time was compared during baseline testing (A), following saline administration (B), and after AMPH treatment (C). Data are presented as means + SEM. ∗p < 0.05, ∗∗p < 0.001. [file Image_1.JPEG]

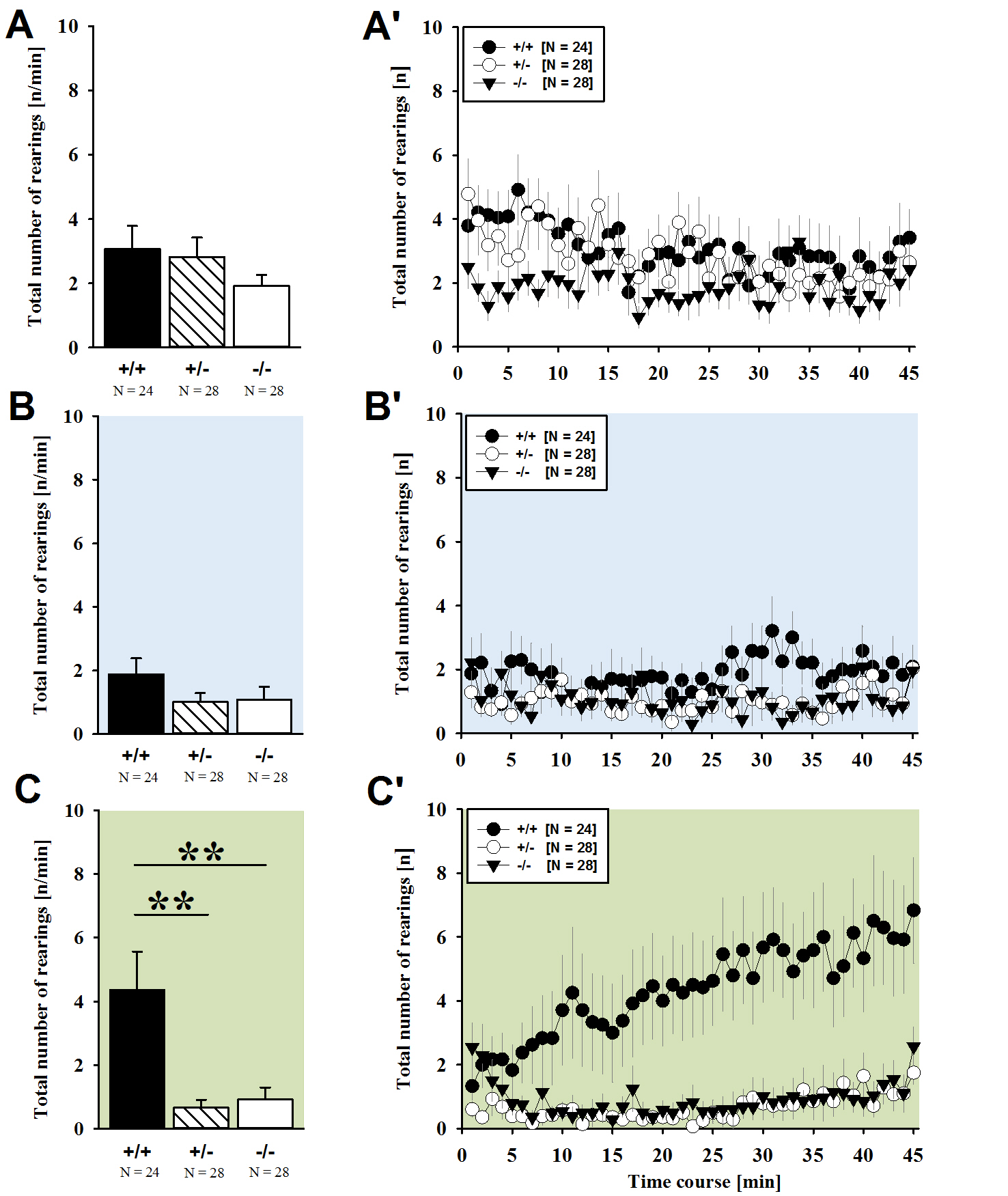

Supplement: FIGURE S2 — AMPH-induced rearing behavior in adult Shank1 mice. (A–C) Bar graphs and (A′–C′) line graphs depicting the rearing behavior by Shank1+/+ (black bar/black circle), Shank1+/- (striped bar/white circle), and Shank1-/- (white bar/black triangle). Rearing behavior was compared during baseline testing (A,A′), following saline administration (B,B′), and after AMPH treatment (C,C′). Data are presented as means + SEM or means ± SEM. ∗∗p < 0.001. [file Image_2.JPEG]

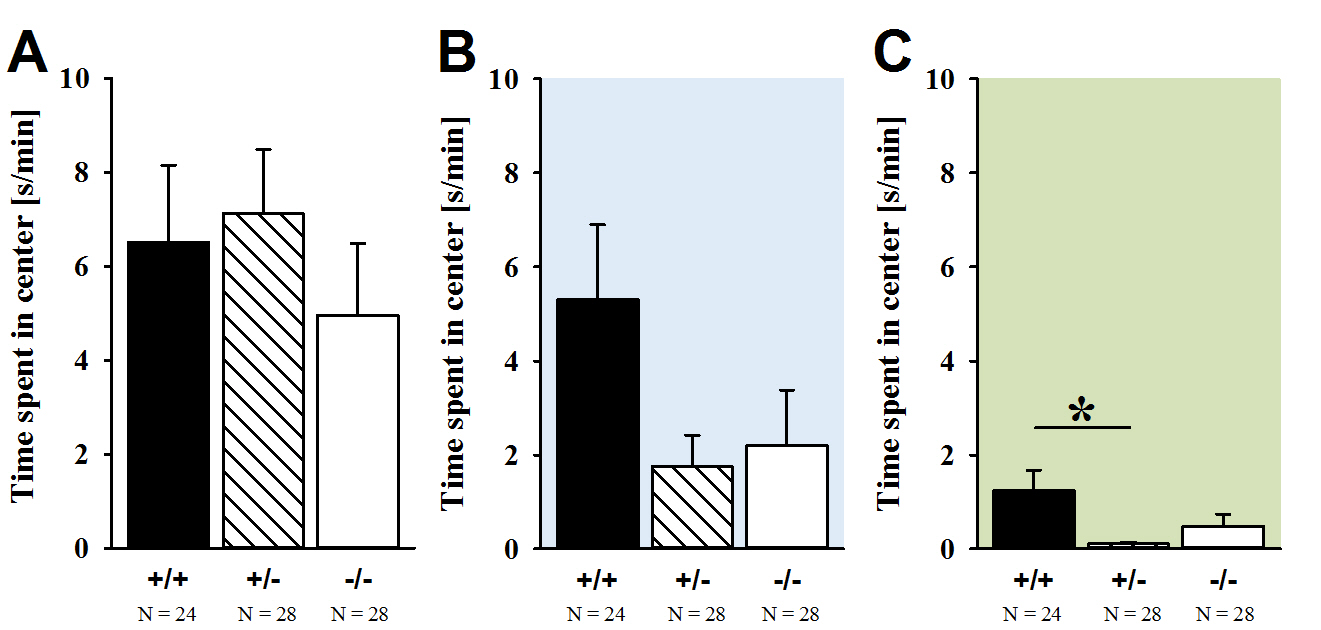

Supplement: FIGURE S3 — Time spent in the center in adult Shank1 mice treated with AMPH. (A–C) Bar graphs depicting the time spent in the center by Shank1+/+ (black bar), Shank1+/- (striped bar), and Shank1-/- (white bar). Center time was compared during baseline testing (A), following saline administration (B), and after AMPH treatment (C). Data are presented as means + SEM. ∗p < 0.05. [file Image_3.JPEG]

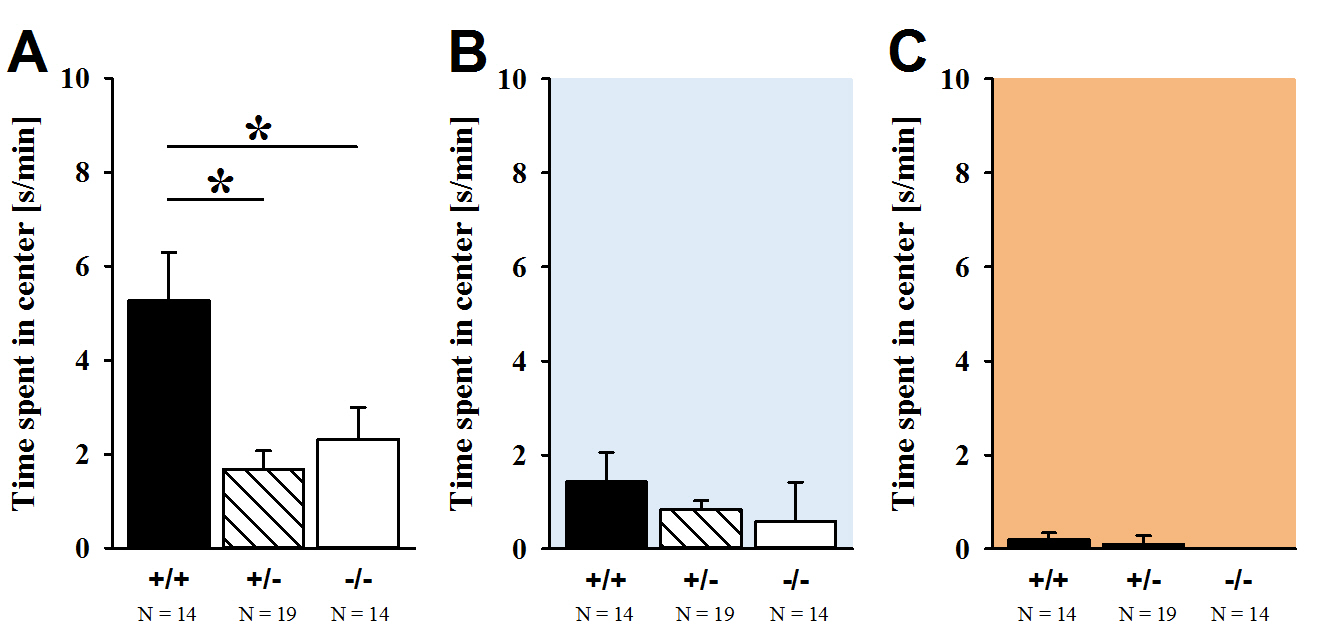

Supplement: FIGURE S4 — Time spent in the center in adult Shank1 mice treated with MDMA. (A–C) Bar graphs depicting the time spent in the center by Shank1+/+ (black bar), Shank1+/- (striped bar), and Shank1-/- (white bar). Center time was compared during baseline testing (A), following saline administration (B), and after MDMA treatment (C). Data are presented as means + SEM. ∗p < 0.05. [file Image_4.JPEG]

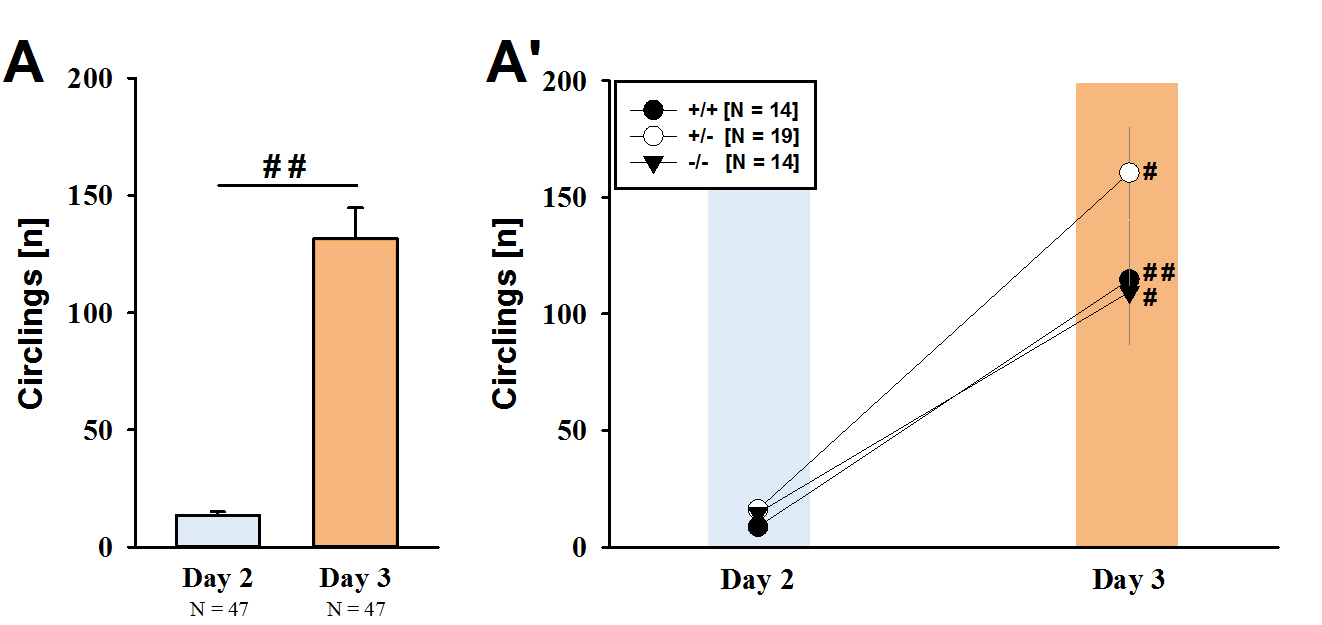

Supplement: FIGURE S5 — MDMA-induced repetitive and stereotyped circling behavior in adult Shank1 mice treated with MDMA. (A) Bar graph depicting the circling behavior displayed by all genotypes following saline administration (blue bar) and after MDMA treatment (orange bar). (A′) Line graph depicting the circling behavior displayed by Shank1+/+ (black circle), Shank1+/- (white circle), and Shank1-/- (black triangle) over two consecutive test days, i.e., following saline administration (blue are) and after MDMA treatment (orange area). Data are presented as means + SEM or means ± SEM. ##p < 0.001 (A), #p < 0.05 and ##p < 0.001 vs. day 2 (A′). [file Image_5.JPEG]
